# Biosorbents for Removing Hazardous Metals and Metalloids †

**DOI:** 10.3390/ma10080857

**Published:** 2017-07-26

**Authors:** Katsutoshi Inoue, Durga Parajuli, Kedar Nath Ghimire, Biplob Kumar Biswas, Hidetaka Kawakita, Tatsuya Oshima, Keisuke Ohto

**Affiliations:** 1 Department of Applied Chemistry, Faculty of Science and Engineering, Saga University, Honjo-machi 1, Saga 840-8574, Japan; kawakita@cc.saga-u.ac.jp (H.K.); ohtok@cc.saga-u.ac.jp (K.O.); 2 Nanomaterial Research Institute, National Institute of Advanced Industrial Science and Technology (AIST), 1-1-1 Higashi, Tsukuba, Ibaragi 305-8565, Japan; parajuli.durga@aist.go.jp; 3 Central Department of Chemistry, Tribhuvan University, Kritipur, Kathmandu, Nepal; knghimire@gmail.com; 4 Department of Chemical Engineering, Faculty of Engineering and Technology, Jessore University of Science and Technology, Jessore 7408, Bangladesh; bipshi2001@yahoo.co.uk; 5 Department of Applied Chemistry, Faculty of Engineering, University of Miyazaki, Gakuen Kibanadai-nishi 1-1, Miyazaki 889-2192, Japan; oshimat@cc.miyazaki-u.ac.jp

**Keywords:** biosorbents, adsorptive removal, aquatic environment, hazardous metals and metalloids

## Abstract

Biosorbents for remediating aquatic environmental media polluted with hazardous heavy metals and metalloids such as Pb(II), Cr(VI), Sb(III and V), and As(III and V) were prepared from lignin waste, orange and apple juice residues, seaweed and persimmon and grape wastes using simple and cheap methods. A lignophenol gel such as lignocatechol gel was prepared by immobilizing the catechol functional groups onto lignin from sawdust, while lignosulfonate gel was prepared directly from waste liquor generated during pulp production. These gels effectively removed Pb(II). Orange and apple juice residues, which are rich in pectic acid, were easily converted using alkali (e.g., calcium hydroxide) into biosorbents that effectively removed Pb(II). These materials also effectively removed Sb(III and V) and As(III and V) when these were preloaded with multi-valent metal ions such as Zr(IV) and Fe(III). Similar biosorbents were prepared from seaweed waste, which is rich in alginic acid. Other biosorbents, which effectively removed Cr(VI), were prepared by simply treating persimmon and grape wastes with concentrated sulfuric acid.

## 1. Introduction

Industrial development has caused serious environmental pollution problems that have negative effects on society. Discharges of toxic heavy metals and metalloids in waste produced by industrial plants (e.g., mineral processing, metal plating, electric, electronic, and chemical plants) cause unacceptable environmental contamination. Conventional treatments of effluents containing heavy metals and metalloids include flotation, coagulation/precipitation, membrane separation, electrochemical treatments, ion exchange and adsorption. Precipitation followed by coagulation has been used extensively because of the simplicity and cheapness of the processes. A typical precipitation method involves adding lime to form water-insoluble metal hydroxides, and such a process removes cationic heavy metals such as Pb(II), Cd(II) and Cu(II). The precipitates are finally dumped at landfill sites. However, such metal hydroxide precipitates contain large amounts of water, so they should be dewatered and dried before being dumped, but this is a costly process. Furthermore, effluents typically contain sulfate, which is nontoxic and does not need to be removed, at a much higher concentration than these heavy metal concentrations, so adding lime also causes large amounts of gypsum to precipitate, increasing the volume of the precipitate to be dumped, and increasing costs. Removal techniques that are very selective for heavy metals and metalloids are therefore required from an economic point of view.

Adsorption techniques, including ion-exchange, are more attractive than precipitation techniques because solid adsorbents can take up metals even at trace concentration using low solid/liquid ratio because solid adsorbents contain numerous micro-pores with very large specific surface areas. However, conventional highly porous adsorbents, such as activated carbon and ion exchange resins (including chelating ion exchange resins), are expensive and suffer another problem from a practical point of view. This problem is that, most effluents contain very fine solid particles that enter and clog the micro-pores (which contain the sites at which heavy metals are adsorbed) of the adsorbent, causing the effectiveness of the adsorbent to be deteriorate.

In recent decades, the interest of the scientific and engineering community in the sequestering of metal ions by living or inactive biomass has increased [1]. Biosorption, especially using biomass waste, is currently considered to be one of the most promising environmentally benign techniques for recovering and removing metals from aqueous solutions. Biosorption-based processes offer a number of advantages including low production cost of adsorbents, simple operation, high efficiency, improved selectivity for specific metals of interest, short operation time, no generation of secondary refractory wastes, easy treatment of spent adsorbents by incineration and so forth. In biosorbents, the chemical components that effectively adsorb metal ions include several kinds of polysaccharide (e.g., pectic acid, alginic acid and chitosan), polyphenol compounds (e.g., tannin and catechin), and proteins. Additionally, biomass with an insignificant adsorption behavior can be changed into an adsorbent with excellent adsorption properties by subjecting them to simple chemical treatments. For example, although chitin poorly adsorbs most metal ions, chitosan that has been subjected to a simple hydration reaction using a concentrated solution of sodium hydroxide has a remarkable ability to adsorb heavy metal ions such as Cu(II) [2,3]. Another example is lignin, a major component of all plants (along with cellulose). Although lignin itself poorly adsorbs metal ions, much better adsorption ability is given by interacting with phenol or polyphenol compounds. This will be mentioned later in detail.

In the present paper, we introduce biosorbents we have prepared and describe their adsorption behaviors for hazardous heavy metals and metalloids.

## 2. Lignophenols

**Scheme 1:**
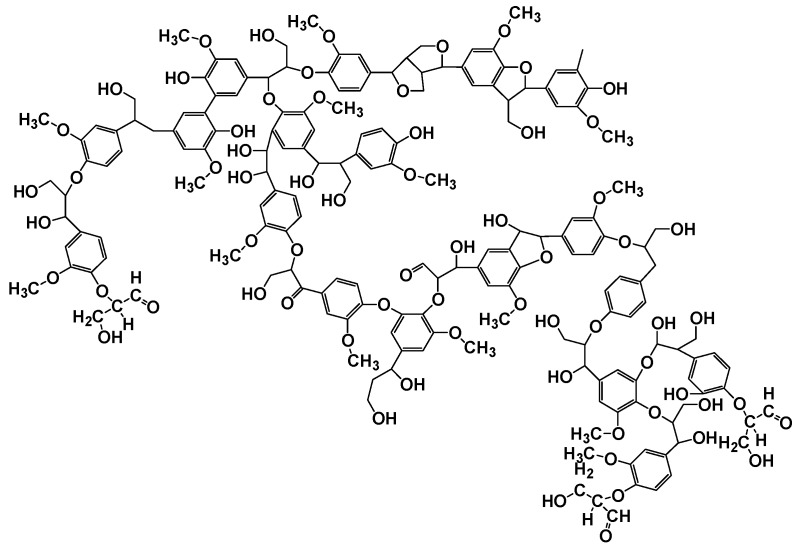
Representative chemical structure of lignin.

Lignin is one of the major components in all plants, the other major components being polysaccharides such as cellulose. Lignin is an amorphous multifunctional phenolic network polymer, an example of which is shown in Scheme 1, containing hydroxyl, ether, and carbonyl groups [4]. Of the functional groups in lignin, only phenolic hydroxyl groups have some affinity for metal ions. However, insignificant adsorption of metal ions occurs because of low content (<10%) of the functional groups of hydroxyl groups in lignin. Additionally, isolating lignin from plant material (i.e., separating lignin from cellulose) is difficult because the lignin polymer units interpenetrate the cellulose structures. However, Funaoka recently developed a simple method for isolating lignin from plant biomass such as wood powder by treating the biomass with phenol or polyphenol compounds (such as catechol) in concentrated sulfuric acid [5]. This process gives novel biodegradable plastics called lignophenols. We have previously attempted to use lignophenols as biosorbents for adsorbing heavy metals [6].

**Scheme 2:**
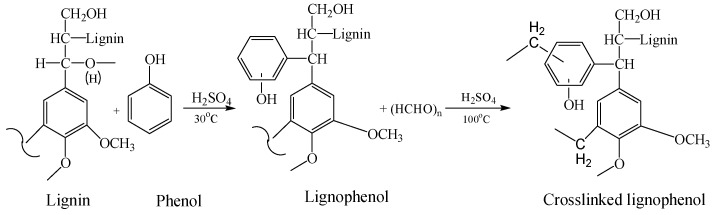
Synthesis and crosslinking of lignophenol.

Lignophenols are partly water soluble because they contain many alcoholic hydroxyl groups. We therefore crosslinked lignophenols by treating them with formaldehyde to prevent the biosorbents from dissolving in aqueous media. The lignophenol synthesis and crosslinking processes are shown in Scheme 2.

Thus, prepared crosslinked lignophenol compounds were examined by scanning electron microscopy. No holes or cracks were found on the surfaces of the materials, and the materials were found to be nonporous.

**Figure 1:**
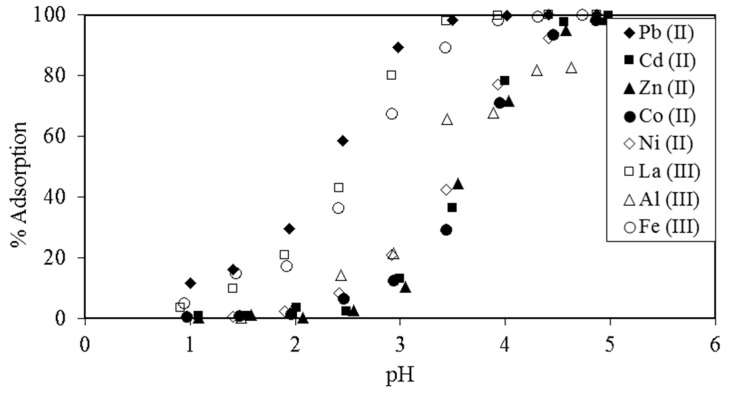
% adsorption of different metal ions by crosslinked lignocatechol gel at different pH values at equilibrium. Initial metal ion concentration = 0.2 mM, dry weight of lignocatechol gel = 20 mg, test solution volume = 15 mL [6]. (With permission for reuse from Elsevier B.V.).

The relationships between the percentages of metal ions in solution that were adsorbed (% adsorption) and the equilibrium pH when some heavy metal cations were exposed to crosslinked lignocatechol gel in a buffer (0.1 M (M = mol/L)) hydrochloric acid and 0.1 M N-[2-hydroxyethyl] piperazine-N’-[2-ethanesulfonic acid] (HEPES), later called HEPES buffer solution, are shown in Figure 1. Here, % adsorption was defined as shown in Equation (1).

% Adsorption = ((Initial metal concentration − Metal concentration at equilibrium)/Initial metal concentration) × 100(1)

It can be seen from this figure that adsorption increased with increasing pH, suggesting that these metal cations were adsorbed through cation exchange with the phenolic hydroxyl groups of the crosslinked lignocatechol gel, which will have released hydrogen ions, as will be discussed in detail later. It can also be seen that the selectivity the for the metal ions that were tested decreased in the order: Pb(II)~La(III) > Fe(III) > Al(III) > Ni(II)~Zn(II)~Cd(II)~Co(II). These results suggest that the different metal ions could be separated by using the crosslinked lignocatecohl gel at specific pH values.

**Figure 2:**
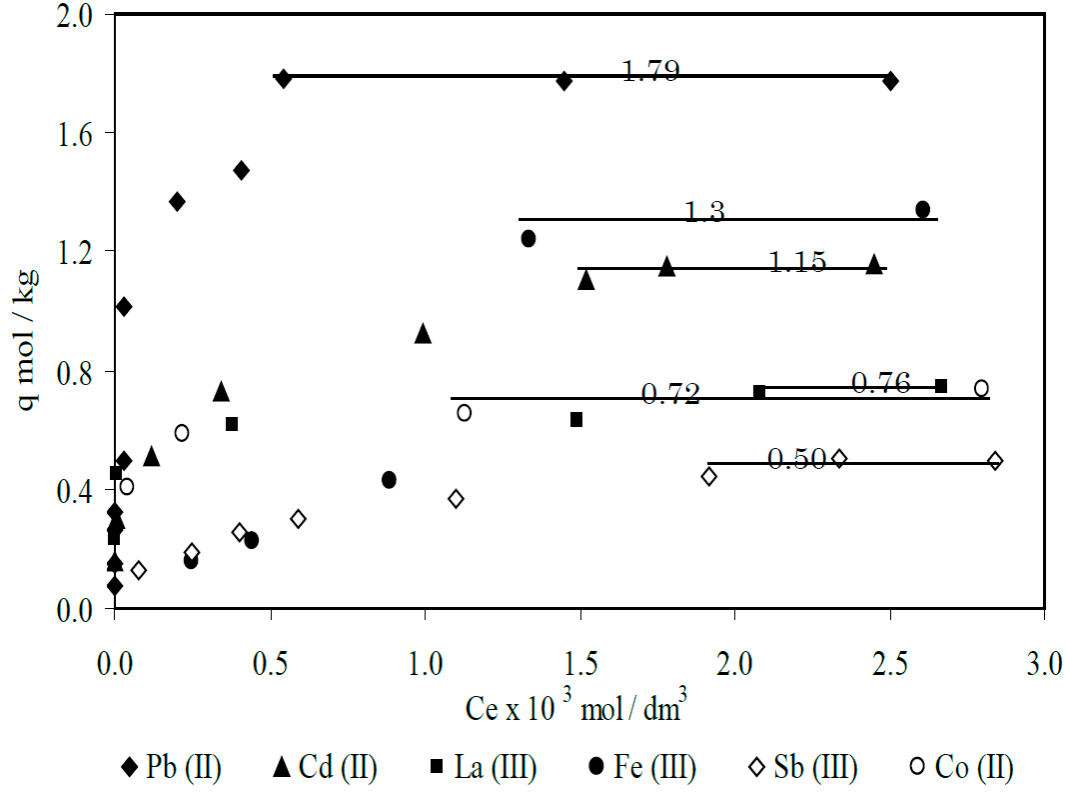
Relationships between the amounts of some metal ions adsorbed from a buffer solution by the crosslinked lignocatechol gel (q) and the equilibrium metal concentrations (Ce), i.e., adsorption isotherms, at 30 °C The Pb(II), Cd(II), La(III), Sb(III), and Co(II) tests were performed at pH = 5.2, and the tests for Fe(III) were performed at pH = 2.5. Dry weight of lignocatechol gel = 20 mg, test solution volume = 15 mL, shaking time = 24 h.

Isotherms for the adsorption of some metal ions by the crosslinked lignocatechol gel are shown in Figure 2. It appears that adsorption of these metal ions followed the Langmuir adsorption model, i.e., the adsorption of these metal ions increased with increasing metal ion concentration at low concentrations but tended to approach a constant value for each metal ion at high metal ion concentrations. The maximum adsorption capacity of the gel for each metal ion was determined from the constant value and is shown in this figure. It is worth noting that this biosorbent had a high adsorption capacity for Pb(II), which is a typical toxic heavy metal ion.

**Scheme 3:**
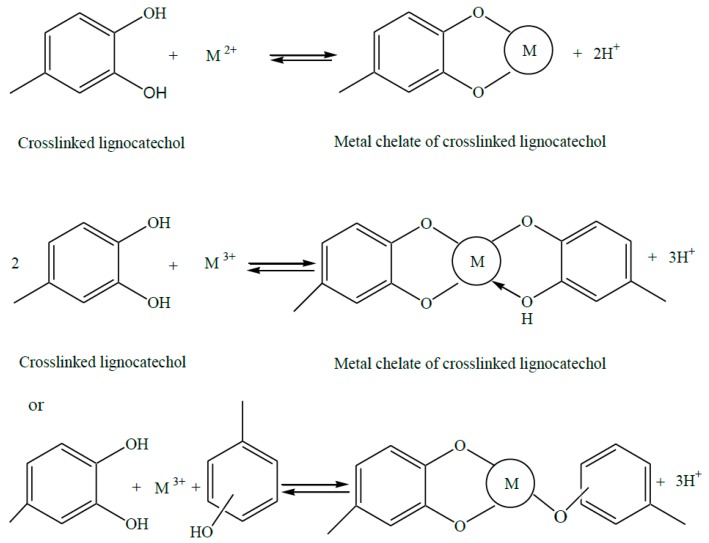
Inferred mechanism through which divalent and trivalent metal ions are adsorbed by crosslinked lignocatechol [6]. (With permission for reuse from Elsevier B.V.).

Natural lignin has relatively few phenolic hydroxyl groups (~10% of the total number of functional groups), so immobilizing catechol ligands onto the lignin polymer matrix greatly improves the adsorption characteristics of the product. The long flexible polymer chains can take any shape required to form stable metal chelates, and this is expected to be an important factor in causing this lignin derivative to have a high adsorption efficiency. A number of cation-exchangeable monophenolic and polyphenolic hydroxyl groups that are naturally present on the original lignin molecule or are immobilized during the chemical treatment described earlier have excellent metal ion adsorption characteristics because of the cation exchange mechanism described below. For example, it can be inferred that divalent and trivalent metal ions are adsorbed through the formation of stable five-membered-ring chelates, as shown in Scheme 3.

**Figure 3:**
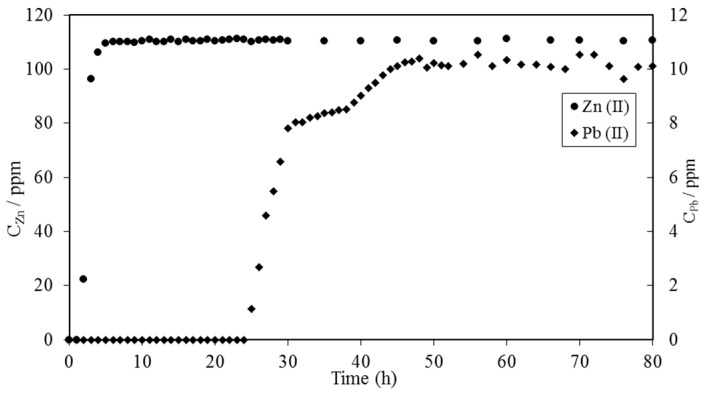
Pb(II) and Zn(II) breakthrough profiles when a solution containing 11 mg/L Pb(II) and 110 mg/L Zn(II) was passed through a column packed with crosslinked lignocatechol. pH = 2.5, weight of adsorbent packed into the column = 0.40 g, flow rate = 3.00 mL/h [6]. (With permission for reuse from Elsevier B.V.).

**Figure 4:**
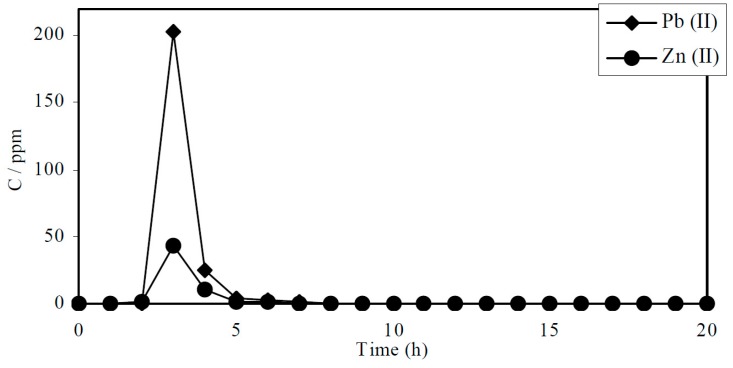
Pb(II) and Zn(II) elution profiles when the loaded crosslinked lignocatechol column was eluted with 1 M hydrochloric acid. Flow rate = 3.00 mL/h [6]. (With permission for reuse from Elsevier B.V.).

The results of the batch-wise adsorption tests mentioned above led us to perform a chromatographic test to separate a small amount of Pb(II) from a large excess of Zn(II) using a column packed with crosslinked lignocatechol gel. The Pb(II) and Zn(II) breakthrough profiles are shown in Figure 3, and the conditions used are described in the legend. It is clear from the figure that Zn(II) broke through immediately after the test solution was applied, because Pb(II) was much more selectively adsorbed than Zn(II) (as shown in Figure 1). The Pb(II) and Zn(II) elution profiles when the loaded column was eluted with 1 M hydrochloric acid solution after Pb(II) breakthrough had finished are shown in Figure 4. Pb(II) was eluted at a high concentration (20 times the concentration in the original test solution). These results suggest that crosslinked lignocatechol can be used to effectively separate Pb(II) from Zn(II) and pre-concentrate the Pb(II).

## 3. Crosslinked Lignosulfonate

**Scheme 4:**
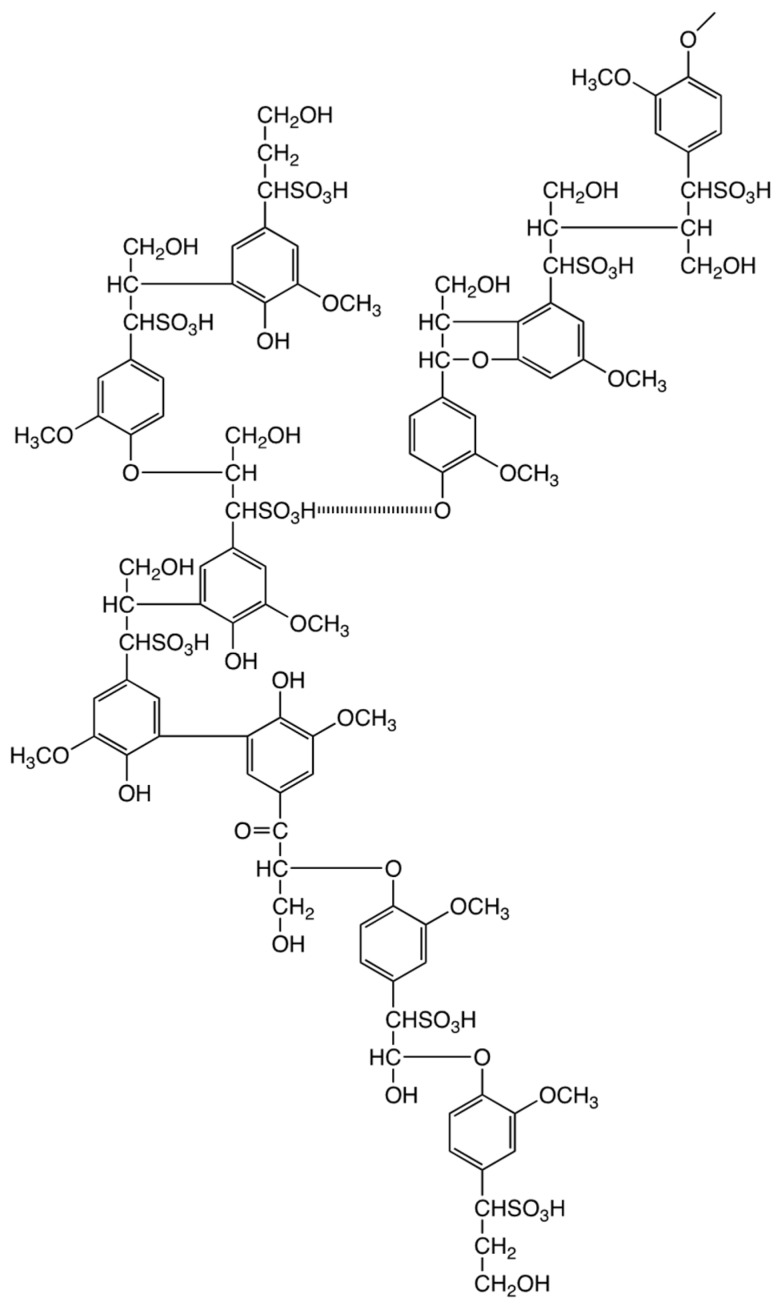
Typical lignosulfonates chemical structure [7].

Lignosulfonates are byproducts of wood pulp production using the sulfite pulping method. Lignosulfonates are water-soluble anionic polyelectrolyte polymers, and a representative chemical structure is shown in Scheme 4 [7]. Lignosulfonates are recovered from spent sulfite pulping liquid (a red or brown liquor) by adding excess calcium hydroxide to cause the lignosulfonates to precipitate. Lignosulfonates have been commercially used in various ways, e.g., as plasticizers when making concrete and to decrease the amount of water required to produce plaster board; but, demands for lignosulfonates is limited and most lignosulfonates are discarded.

It can be seen from the chemical structure that lignosulfonates contain many sulfonic acid functional groups, so it could be expected that lignosulfonates could be used as cation exchange materials. We therefore attempted to prepare a new biosorbent for removing heavy metals from solution by crosslinking lignosulfonates using paraformaldehyde [8].

**Figure 5:**
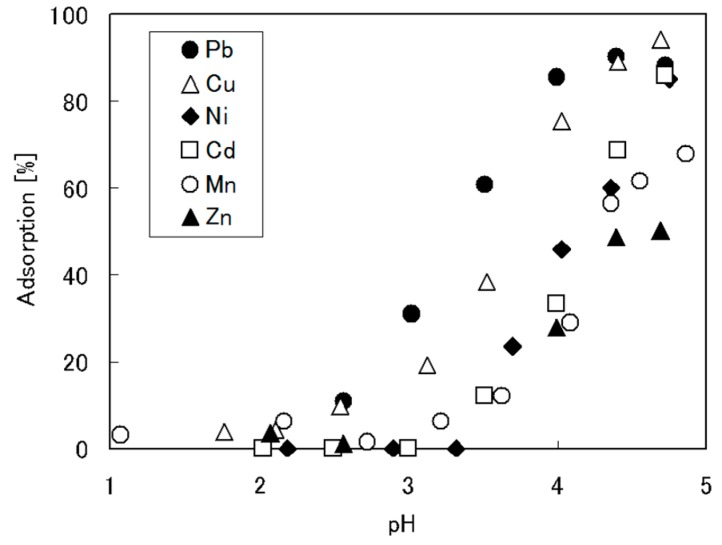
Adsorption of some divalent metal ions by crosslinked lignosulfonate gel at different equilibrium pH values. Initial metal ion concentration = 15 mg/L, dry weight of gel = 10 mg, test solution volume = 7.5 mL [8]. (With permission for reuse from The Society of Chemical Engineers, Japan).

The % adsorption of some divalent metal ions adsorbed from a HEPES buffer solution by the crosslinked lignosulfonate gel are plotted against equilibrium pH of the solution at 30 °C in Figure 5. The adsorption increased with increasing pH similar to the case for lignocatechol gel, suggesting that the metal ions were adsorbed through cation exchange with the sulfonic acid functional groups, (one of the main functional groups of this biosorbent), releasing hydrogen ions. Including trivalent metal ions and Fe(II), which are not shown in Figure 5, the selectivity for the metal ions that were tested decreased in the order: La(III) > Pb(II)~Fe(III) > Fe(II)~Cu(II)~Al(III) > Cd(II)~Ni(II) > Mn(II)~Zn(II). It is worth noting that, similar to the case of lignocatechol gel, the crosslinked lignosulfonates gel was more selective for Pb(II) than the other metal ions.

Adsorption isotherms indicated that La(III), Cu(II), and Pb(II) were adsorbed according to the Langmuir’s adsorption isotherm model. The maximum amount of La(III), Cu(II), and Pb(II) that were adsorbed were 0.47, 0.86 and 0.76 mol/kg, respectively. The maximum amount of Pb(II) that was adsorbed by the lignosulfonate gel was lower than the maximum amount adsorbed by the lignocatechol gel. However, Pb(II) and Zn(II) were separated well using a column packed with the lignosulfonate gel.

## 4. Crosslinked Pectic Acid and Alginic Acid

**Scheme 5:**
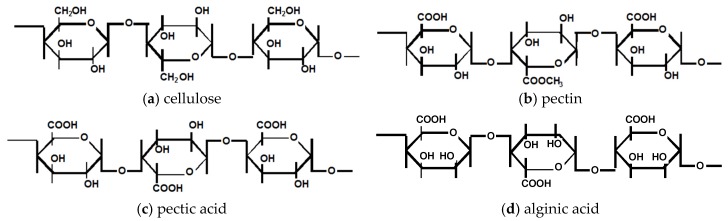
Chemical structures of cellulose, pectin, pectic acid and alginic acid.

Pectic acid and alginic acid are typical acidic polysaccharides. The chemical structures of cellulose, pectin, pectic acid and alginic acid are shown in Scheme 5.

Pectin is contained in fruit, particularly oranges and apples, and acts as a cement between cells. Pectin is a weakly acidic polysaccharide, which is basically pectic acid with some methyl-esterified carboxylic acid functional groups. These methyl esters can be easily hydrolyzed using an alkali such as sodium hydroxide or calcium hydroxide, converting the pectin into a pectic acid salt. Calcium salts of alginic acid are major components of seaweed. Pectic acid and alginic acid contain carboxylic acid functional groups, so they are expected to function as weakly acidic cation exchange materials. We therefore attempted to prepare new biosorbents for removing heavy metals from solution by crosslinking pectic acid and alginic acid using epichlorohydrin [9].

**Figure 6:**
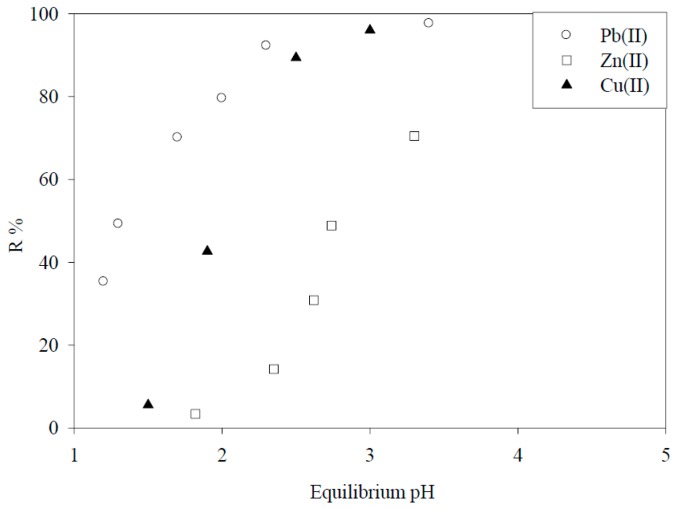
Adsorption of Pb(II), Zn(II), and Cu(II) by crosslinked pectic acid as a function of the equilibrium pH [9]. (With permission for reuse from Elsevier B.V.).

**Figure 7:**
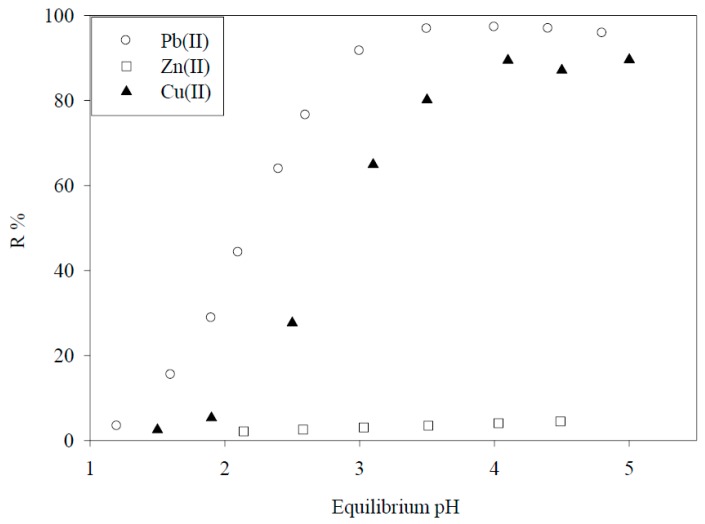
Adsorption of Pb(II), Zn(II) and Cu(II) by crosslinked alginic acid as a function of the equilibrium pH values [9]. (With permission for reuse from Elsevier B.V.).

**Figure 8:**
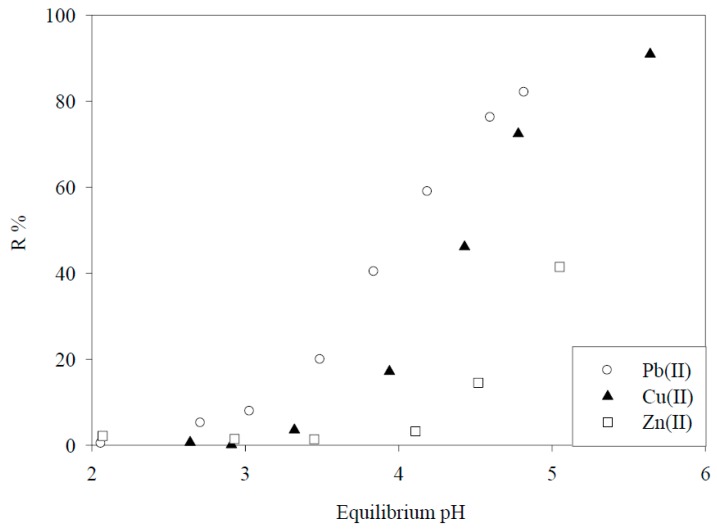
Adsorption of Pb(II), Zn(II) and Cu(II) by DIAION WK11 resin as a function of the equilibrium pH [9]. (With permission for reuse from Elsevier B.V.).

The percentages of Pb(II), Zn(II), and Cu(II) adsorbed (R) by crosslinked pectic acid and alginic acid are plotted against the equilibrium pH in Figure 6 and Figure 7, respectively. As can be seen, Pb(II) was selectively and strongly adsorbed in preference to Zn(II) and Cu(II) by both adsorbents over the whole pH range that was tested. It can be seen from Figure 8 that DIAION WK11 resin, a commercially available weakly acidic cation exchange resin containing carboxylic acid functional groups, gave similar results. However, this resin separated Pb(II) from Zn(II) and Cu(II) less effectively than did the crosslinked pectic and alginic acid gels. Additionally, this resin adsorbed the metal ions at a much higher pH than did the crosslinked pectic acid and alginic acid gels, suggesting that the synthetic resin has a much weaker ion exchange ability or adsorptive power for cationic species than do the crosslinked pectic acid and alginic acid gels.

Adsorption isotherm tests of Pb(II) were performed at 30 °C using the crosslinked pectic acid and alginic acid gels and WK11 resin, and it was found that adsorption followed the Langmuir adsorption model for all the adsorbents. The maximum adsorption capacities of the crosslinked pectic acid, crosslinked alginic acid, and WK11 resin were 1.6, 1.2 and 0.98 mol/kg, respectively. The maximum adsorption capacity of the crosslinked pectic acid gel was much higher than the maximum adsorption capacity of the synthetic ion exchange resin, and the maximum adsorption capacity of the alginic acid gel was a little higher than the maximum adsorption capacity of the synthetic ion exchange resin.

**Scheme 6:**
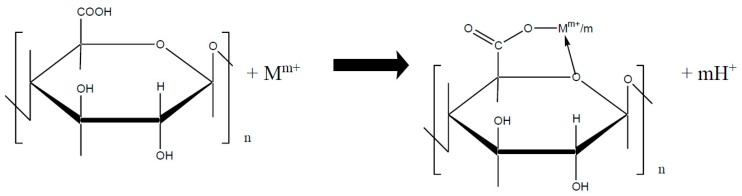
Inferred mechanism for the adsorption of m-valent metal cations by crosslinked pectic acid [9] (the mechanism for crosslinked alginic acid is the same). (With permission for reuse from Elsevier B.V.).

As is shown in Figure 6, Figure 7 and Figure 8, the adsorption of metal cations increased with increasing pH similar to the results for the crosslinked lignocatechol and lignosulfonate mentioned earlier. This suggests that adsorption by the crosslinked pectic acid and alginic acid gels and WK11 resin occurs through cation exchange between the carboxylic functional groups and metal cations, releasing hydrogen ions. However, the oxygen atoms on the pyranose rings of the crosslinked pectic acid and alginic acid gels play important roles in the adsorption of metal cations, cooperating with the carboxylic groups to form stable five-membered ring chelates as shown in Scheme 6. This is the main reason that adsorption occurs at much lower pH values and more strongly for acidic polysaccharide gels than for weakly acidic cation exchange resins that contain the same carboxylic functional groups. In addition, these polysaccharides biosorbents have more flexible steric structures than do weakly acidic cation exchange resins, allowing the biosorbents to take configurations suitable for the formation of stable metal chelates.

**Figure 9:**
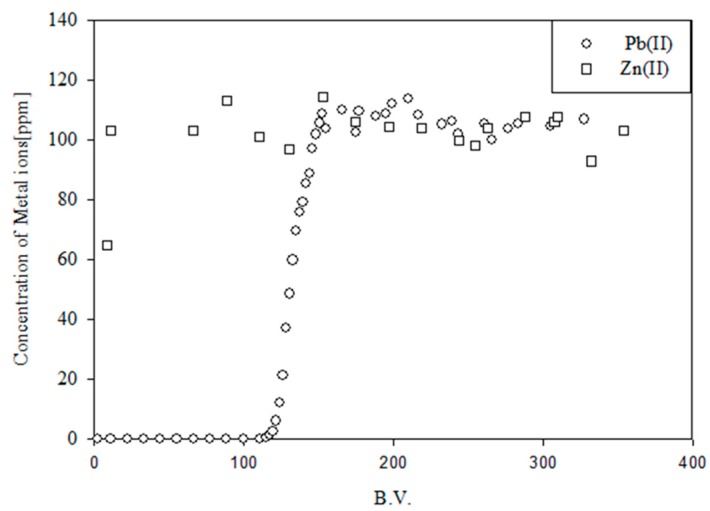
Pb(II) and Zn(II) breakthrough profiles when a solution containing 100 mg/L Pb(II) and 110 mg/L Zn(II) at pH 2.01 was passed through a column packed with crosslinked pectic acid gel. The flow rate was 6.5 mL/h [9]. (With permission for reuse from Elsevier B.V.).

The good separation of Pb(II) and Zn(II) achieved using the crosslinked acidic polysaccharide gels led us to perform separation tests using columns packed with these gels. Pb(II) and Zn(II) breakthrough profiles found when a solution containing 100 mg/L Pb(II) and 100 mg/L Zn(II) was passed through a column packed with crosslinked pectic acid gel are shown in Figure 9. In this and other figures, B.V. (on the horizontal axis) is the bed volume, defined by Equation (2).

B.V. = total volume of solution passed through the column/volume of adsorbent packed into the column(2)

**Figure 10:**
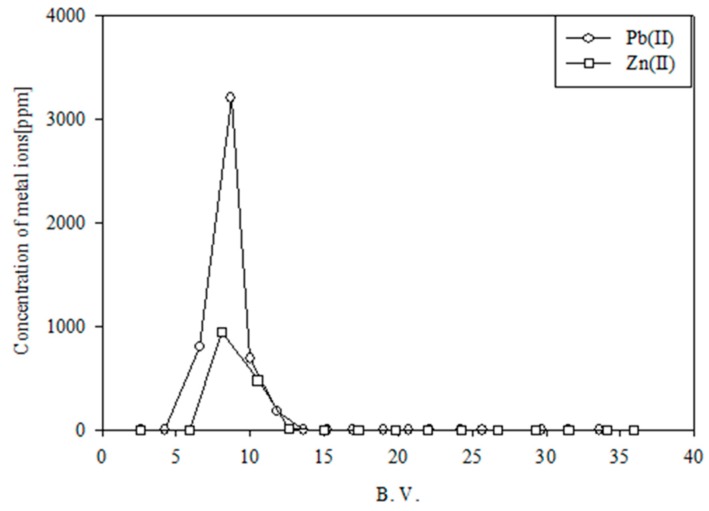
Pb(II) and Zn(II) elution profiles when the column loaded with Pb(II) and Zn(II) in the breakthrough test described in Figure 9 (after complete breakthrough of both metal ions had occurred) was eluted with 1 M nitric acid [9]. (With permission for reuse from Elsevier B.V.).

As can be seen from Figure 9, Pb(II) breakthrough occurred at 115 B.V. and Zn(II) breakthrough occured at 11 B.V., suggesting that Pb(II) and Zn(II) could be easily separated using a column packed with crosslinked pectic acid gel. The adsorbed Pb(II) was completely eluted at 14 B.V., and the concentration in the 1 M nitric acid eluate was 30 times higher than the concentration in the original solution, whereas only a trace of Zn(II) was found in the eluate, as shown in Figure 10.

**Scheme 7:**
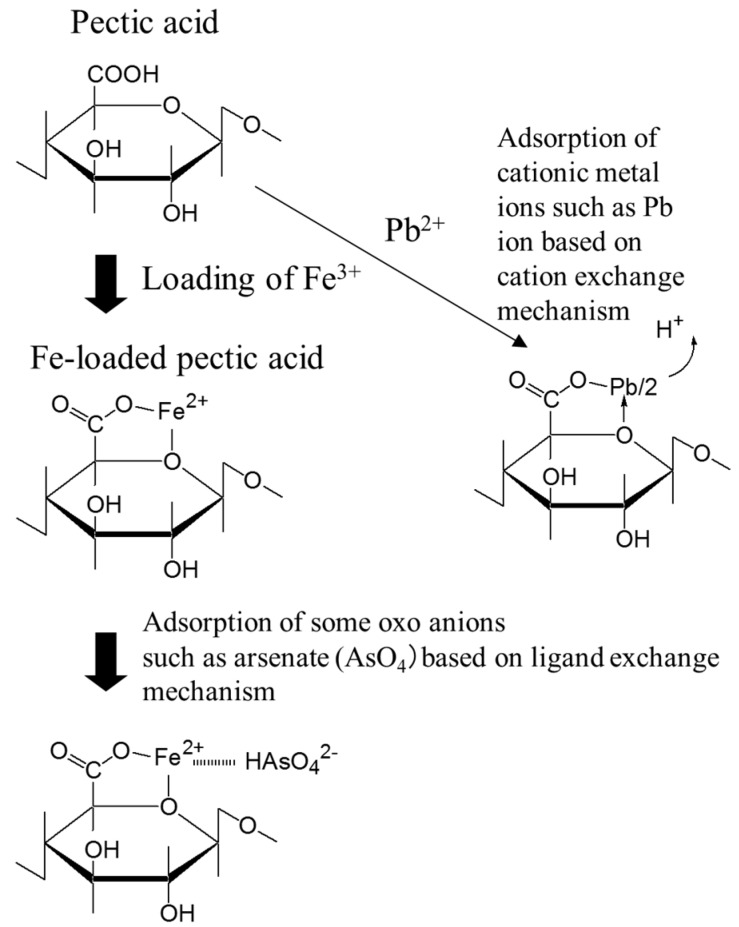
Mechanisms through which heavy metal cations such as Pb(II) are adsorbed by the crosslinked pectic acid gel through cation exchange and through which heavy metal anions and metalloids such as As(V) are adsorbed by crosslinked pectic acid gel loaded with multi-valent metal ions such as Fe(III) through anion exchange [11]. (With permission for reuse from The Mining and Materials Processing of Japan).

Many hazardous heavy metals and metalloids are found in the environment as oxo-anionic species such as AsO_4_^3−^. It is difficult to separate these oxo-anionic species from other anionic species such as sulfate, which are also usually present in environmental media, using conventional anion exchange materials because of the well-known Hoffmeister anion exchange principle series related to the hydration energies of anionic species [10]. Oxo-anionic species have been removed using cation exchange resins loaded with multi-valent metal ions such as Fe(III), which have strong affinities for oxo-anionic species. As mentioned earlier, crosslinked pectic acid and alginic acid gels have strong affinities for multi-valent metal ions such as Fe(III), so we attempted to use these biosorbents loaded with multi-valent metal ions to selectively remove hazardous oxo-anionic species. The concept is shown schematically in Scheme 7 [11].

**Figure 11:**
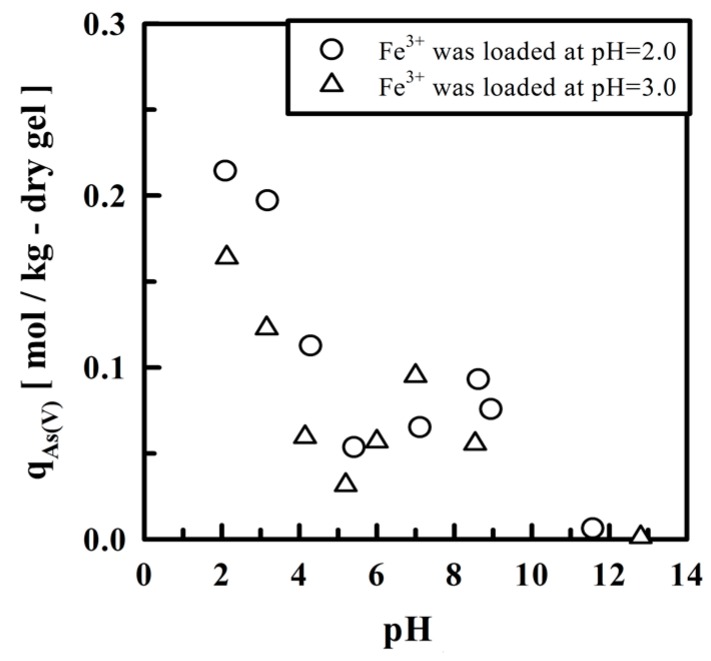
Amount of As(V) adsorbed (q_As(V)_) by crosslinked pectic acid gel loaded with Fe(III) plotted against the equilibrium pH [12]. (With permission for reuse from The Society of Chemical Engineers, Japan).

**Figure 12:**
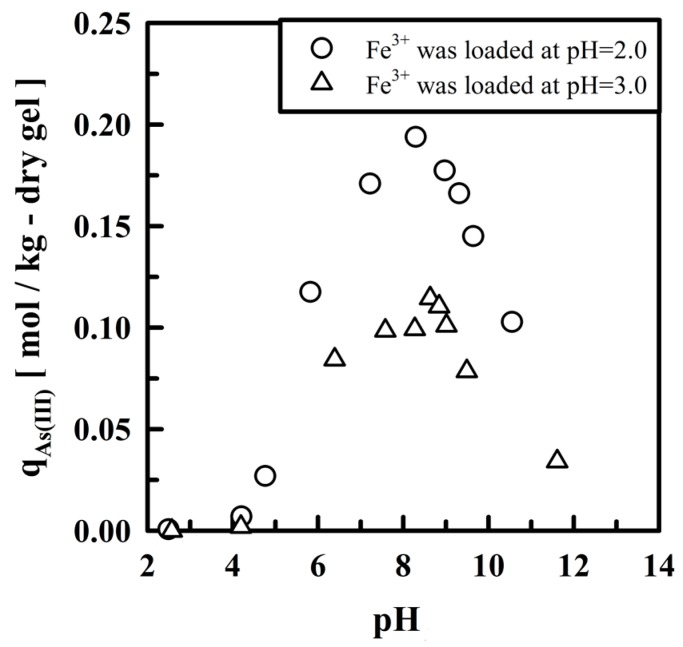
Amount of As(III) adsorbed (q_As(III)_) by crosslinked pectic acid gel loaded with Fe(III) plotted against the equilibrium pH [12]. (With permission for reuse from The Society of Chemical Engineers, Japan).

The amounts of As(V) and As(III) adsorbed by crosslinked pectic acid gel loaded with Fe(III) at pH 2.0 and 3.0 are shown in Figure 11 (As(V)) and Figure 12 (As(III)) [12]. The gels loaded with Fe(III) adsorbed As(V and III) more effectively at pH 2.0 than at pH 3.0, indicating that Fe(III) was easily hydrolyzed and dissolved at pH 3.0 decreasing the amount of As(V and III) that was adsorbed. Adsorption of both As(V) and As(III) was not affected by a high concentration of sulfate or chloride.

## 5. Biosorbents Prepared from Orange and Apple Juice Residues

**Scheme 8:**
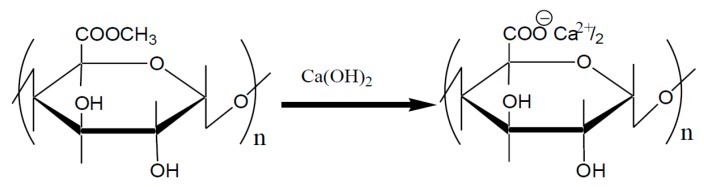
Saponification of pectin using calcium hydroxide.

As mentioned earlier, heavy metals and metalloids are very effectively adsorbed by crosslinked pectic acid and alginic acid gels, which have interesting adsorption characteristics. However, pure pectic acid and alginic acid are expensive, so it may be impractical to use them on a commercial scale. We therefore attempted to prepare biosorbents that could effectively and cheaply remove heavy metals and metalloids from solution using orange and apple juice residues, which are biomass waste products of the fruit juice industry, that contain large amounts of pectic acid [13,14]. However, pectic acid is present in orange and apple juice residues as pectin, which is partly methyl-esterified pectic acid, and has the chemical structure shown in Scheme 5. The pectin in orange and apple juice residues therefore needed to be saponified or hydrated using an alkaline material such as calcium hydroxide to improve its adsorption properties as shown in Scheme 8. The saponified orange and apple juice residue biosorbents prepared in this way are abbreviated as SOJR (Saponified Orange Juice Residue) and SAJR (Saponified Apple Juice Residue), respectively, hereafter.

### 5.1. Removal of Hazardous Metal Cations Using Adsorption Gels Prepared from Orange and Apple Juice Residues

**Figure 13:**
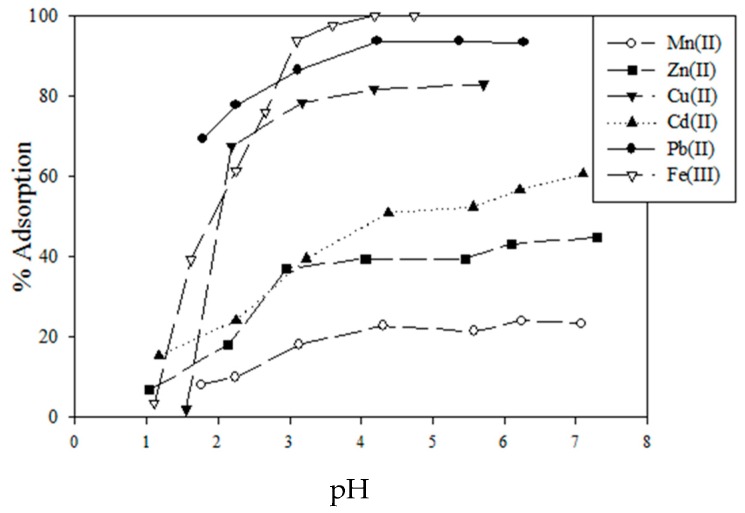
% adsorption (A) of some metal ions by SOJR at different equilibrium pH values [14]. (Copyright 2003 by The Minerals, Metals & Materials Society. Reprinted with permission).

**Figure 14:**
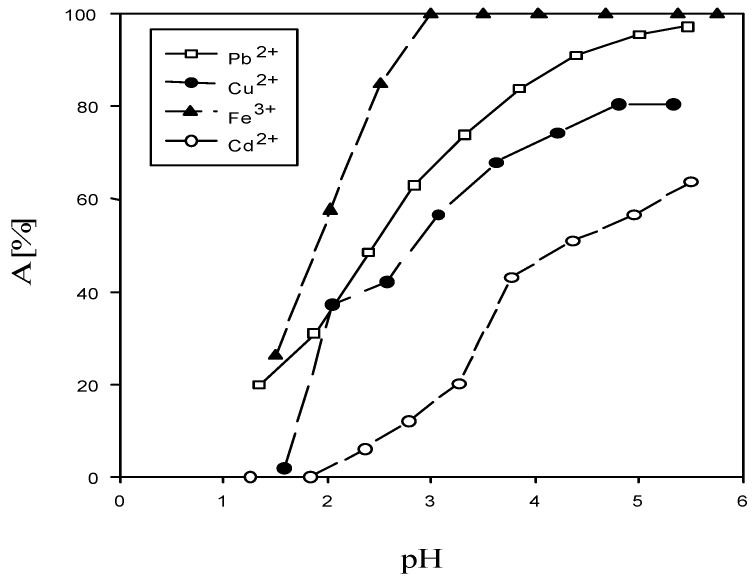
% adsorption (A) of some metal ions by SAJR at different equilibrium pH values [14]. (Copyright 2003 by The Minerals, Metals & Materials Society. Reprinted with permission).

The % adsorption of some metal cations from a HEPES buffer solution by SOJR and SAJR gels are plotted against the equilibrium pH in Figure 13 and Figure 14, respectively. The % adsorption increased with increasing pH, suggesting that adsorption occurred through cation exchange. As can be seen from Figure 13, the selectivity of the SOJR for the metal ions decreased in the order: Pb(II) > Fe(III) > Cu(II) > Cd(II) > Zn(II) > Mn(II) at pH values lower than 3, but adsorption of Fe(III) is the highest at pH values higher than 3. The selectivity of the SAJR for the metal ions decreased in the order: Fe(III) > Pb(II) > Cu(II) > Cd(II). Both biosorbents were relatively selective for Pb(II), similar to the results for crosslinked pectic acid and alginic acid gels. This means that the good selectivity for Pb(II) over other metal ions found for crosslinked pectic acid gel was maintained by the SOJR and SAJR biosorbents.

**Figure 15:**
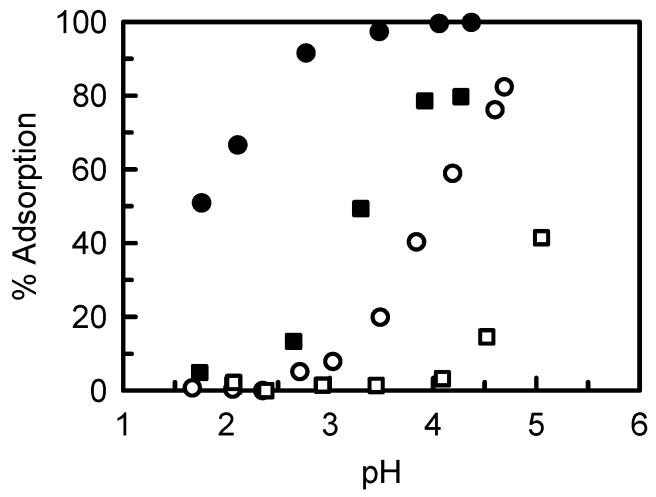
Adsorption of Pb(II) and Zn(II) by SOJR and by WK11 resin [14]. (Copyright 2003 by The Minerals, Metals & Materials Society. Reprinted with permission). ● Pb(II)—SOJR, ▪ Zn(II)—SOJR, ○ Pb(II)—WK11, □ Zn(II)—WK11.

Adsorption of Pb(II) and Zn(II) by SOJR and DIAION WK11 resin, a commercially available weakly acidic cation exchange resin that contains carboxylic acid functional groups, were compared as we did for the crosslinked pectic and alginic acid gels, and the results are shown in Figure 15. It can be seen that adsorption occurred at much lower pH values for SOJR than for WK11 resin, suggesting that Pb(II) and Zn(II) were adsorbed more strongly by SOJR than by WK11 resin. Furthermore, it appeared that the difference between the pH values at which Pb(II) and Zn(II) were adsorbed was greater for SOJR than for WK11 resin, suggesting that these metal ions could be more easily separated using SOJR than using WK11. These results indicate that biosorbents prepared cheaply from biomass waste have better adsorption properties than do synthetic adsorbents produced from petroleum.

**Figure 16:**
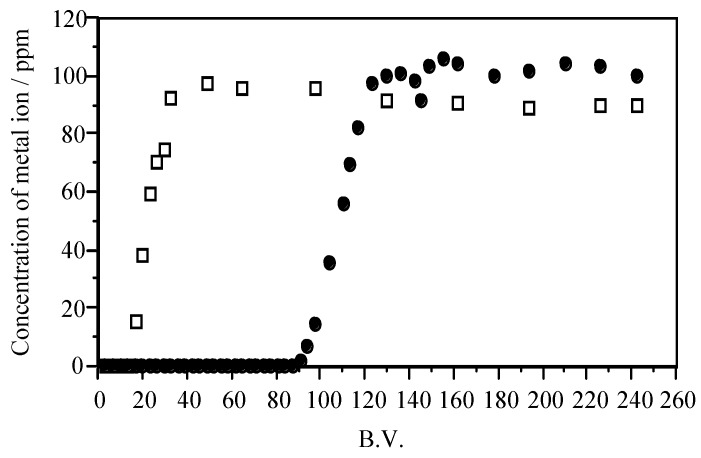
Pb(II) and Zn(II) breakthrough profiles for a column packed with SOJR gel. The Pb(II) and Zn(II) concentrations in the solution passed through the column were 102 and 96 mg/L, respectively, and the pH was 2.6. Flow rate = 5.5 mL/h, dry weight of SOJR gel = 100 mg [14]. (Copyright 2003 by The Minerals, Metals & Materials Society. Reprinted with permission).

**Figure 17:**
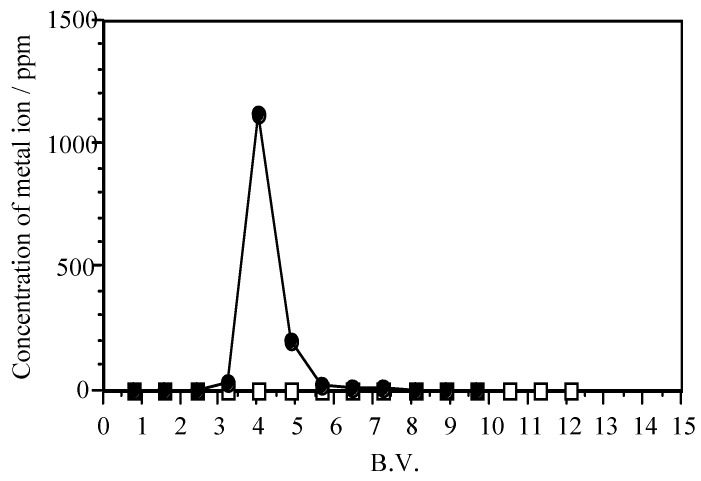
Pb(II) and Zn(II) elution profiles for a column saturated with Pb(II) ions and eluted with 1 M nitric acid solution at a flow rate of 5.5 mL/h [14]. (Copyright 2003 by The Minerals, Metals & Materials Society. Reprinted with permission).

The suggestion from the results of the batch-wise experiments mentioned above that SOJR gel was very selective for Pb(II) over Zn(II) led us to attempt to separate Pb(II) from Zn(II) using a column packed with SOJR gel. The Pb(II) and Zn(II) breakthrough profiles are shown in Figure 16. It can be seen that Pb(II) was satisfactorily separated from Zn(II), and that the results were similar to the results found using a column packed with crosslinked pectic acid gel shown in Figure 9. This suggested that SOJR gel and crosslinked pectic acid gel had similar properties as mentioned earlier. The elution profiles after the gel had been completely saturated with these metal ions, shown in Figure 17, were similar to the profiles shown in Figure 10. It can be seen that Pb(II) was eluted at a concentration about 10 times higher than the concentration in the original solution and that Zn(II) was not detected in the eluate.

**Figure 18:**
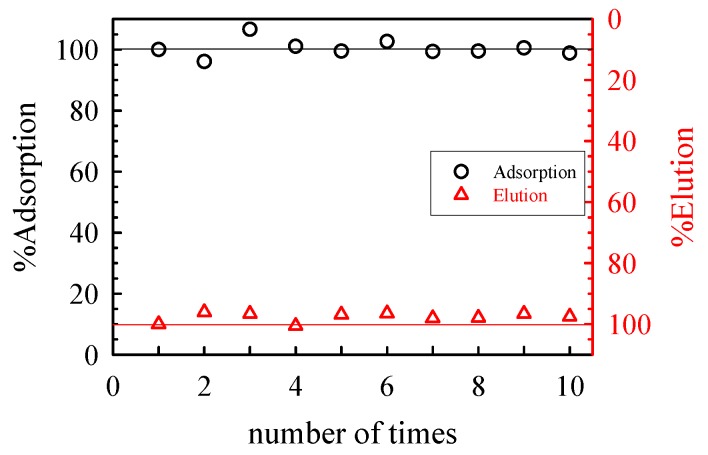
% adsorption and % elution of Pb(II) when a column packed with SOJR gel was repeatedly loaded with Pb(II) and eluted.

The % adsorption by and % elution of Pb(II) from a column packed with SOJR gel when the column was repeatedly loaded and eluted are shown in Figure 18. It can be seen that 100% of the Pb(II) was adsorbed and eluted when the column was used 10 times, suggesting that SOJR is very durable.

**Figure 19:**
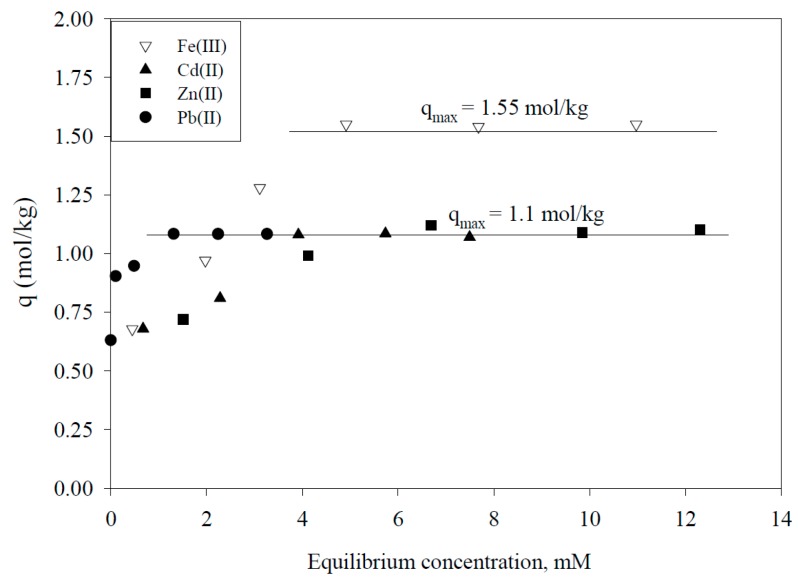
Isotherms for the adsorption of some metal ions by SOJR gel at pH ca. 2 [13]. (With permission for reuse from Elsevier B.V.).

Figure 19 shows the adsorption isotherms of Fe(III), Cd(II), Zn(II) and Pb(II) on SOJR gel. The isotherms suggested that these metal ions are adsorbed through the Langmuire-type adsorption, as was found for other biosorbents mentioned earlier. The maximum Fe(III) adsorption capacity of the SOJR gel determined from the adsorption isotherms was 1.55 mol/kg, and the maximum adsorption capacities for the other three metal ions were all 1.10 mol/kg.

### 5.2. Removing As and Sb Using Adsorption Gels Prepared from Orange Juice Residue

We attempted to remove As(III and V) and Sb(III and V) from test solutions using SOJR gel loaded with various multi-valent metal ions, as we did with crosslinked pectic acid gel. Serious environmental problems are being caused by As(III and V) around the world, particularly in Bangladesh and eastern India. As(III and V) have conventionally been removed from water using the coprecipitation method, using Fe(III). In this method, Fe(III) is added as a chloride or sulfate to a solution containing As(III and V), and the pH of the solution is increased by adding an alkaline material, such as lime. A precipitate of ferric hydroxide forms, and As(III and V) is coprecipitated with the ferric hydroxide. This conventional method suffers some problems, particularly the expense involved in dewatering and drying the precipitate sludge, which contains large amounts of water, and the space required to safely keep the large volumes of sludge for a long time. New economical technologies that do not suffer these problems are required as soon as possible.

**Figure 20:**
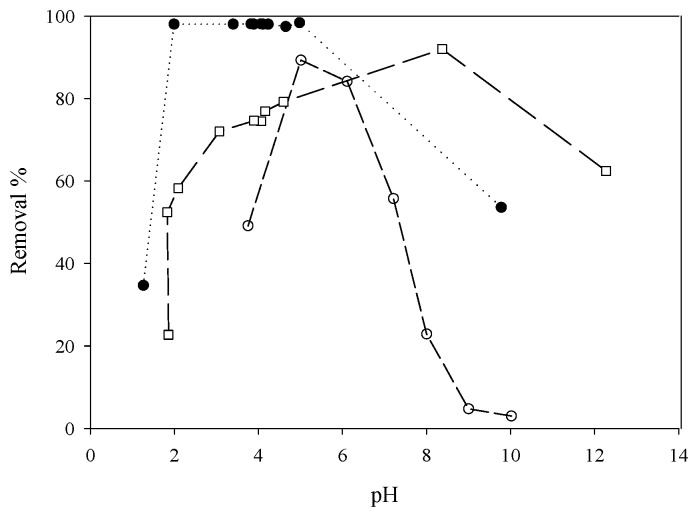
% removal of As through adsorption by iron(III)-loaded SOJR gel and through the conventional coprecipitation method using ferric chloride plotted against equilibrium pH values. ●: Adsorptive removal of As(V) by Fe(III)-loaded SOJR gel. □: adsorptive removal of As(III) by Fe(III)-loaded SOJR gel, ○: removal of As(V) through coprecipitation using ferric chloride [15]. (With permission for reuse from The Mining and Materials Processing Institute of Japan).

Figure 20 shows the plots of % removal of As (V and III) from a synthetic test solution by adsorption using Fe(III)-loaded SOJR gel and using the conventional coprecipitation method using ferric chloride at different equilibrium pH values [15]. It can be seen that Fe(III)-loaded SOJR gel almost completely removed As(V) over a wide pH range (pH 2–6) and removed more than 80% of the As(III) at high pH value (pH 8–10). The conventional coprecipitation method only removed more than 80% of the As(V) in a narrow pH range (pH 5–6) [15].

**Figure 21:**
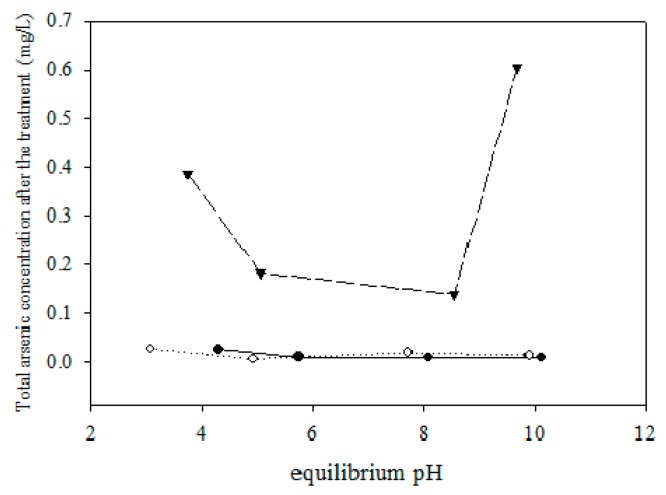
Total As concentrations remaining in mine-water effluent from Horobetsu mine treated using SOJR gel (●), seaweed gel (◇) and the conventional coprecipitation method (▼) (in which ferric hydroxide was precipitated from the acidic mine water containing large amounts of iron when magnesium oxide was added) [15]. (With permission for reuse from The Mining and Materials Processing Institute of Japan).

Figure 21 shows the plots of the total As concentrations remaining in effluent from a closed sulfur mine at Horobetsu, Hokkaido, Japan, treated with the SOJR gel, seaweed gel, which will be mentioned later, and using the coprecipitation method at different equilibrium pH values [15]. The pH of the effluent can be as low as pH 1.8, because it is a typical acid drainage from a closed mine. The total Fe, total As and SO_4_^2−^ concentrations in the effluent were 371, 10.0 and 2510 mg/L, respectively. The biosorbents added to the effluent were not loaded with Fe(III) in advance because they were spontaneously became loaded with Fe(III) present in the effluent during the treatment. No trace of As was found in effluent treated with the SOJR, but the conventional coprecipitation method was ineffective at removing As over the whole pH range that was tested. These results suggest that As can be more effectively adsorbed by a biosorbent prepared from biomass waste than by ferric hydroxide when the conventional method is used.

As mentioned above, Fe(III)-loaded SOJR gel had excellent properties in terms of removing As(III and V) through adsorption. We therefore conducted the adsorption tests in which SOJR gel loaded with various multi-valent metal ions (Ce(III), La(III) and Zr(IV)) was used to remove As(III and V) from a HEPES buffer solution [16,17,18].

**Figure 22:**
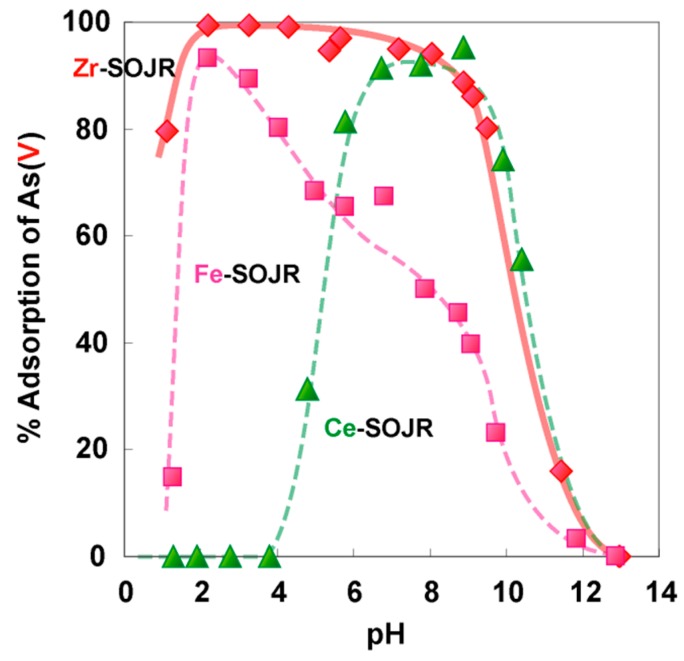
% adsorption of As(V) by SOJR gel loaded with Fe(III), Ce(III) and Zr(IV) at different equilibrium pH values [16]. (With permission for reuse from Indonesian Chemical Society).

Figure 22 shows the plot of % adsorption of As(V) by three types of SOJR gel (loaded with Fe(III), Ce(III) and Zr(IV)) at different equilibrium pH values. The maximum % adsorption by Fe(III)-loaded SOJR gel was found at pH around 2, whereas the maximum % adsorption by Ce(III)-loaded SOJR gel was found at neutral or weakly basic pH values (around pH 8). In other words, the Fe(III)- and Ce(III)-loaded gels most effectively adsorbed As(V) at different pH values or As(V) adsorption was dependent on the metal ion loaded onto the gel. The Zr(IV)-loaded SOJR gel almost quantitatively removed As(V) (higher than 90%) over a wide pH range (pH = 2–8), suggesting that this gel is more suitable than the Fe(III)- and Ce(III)-loaded gels for removing As(V) from water.

**Figure 23:**
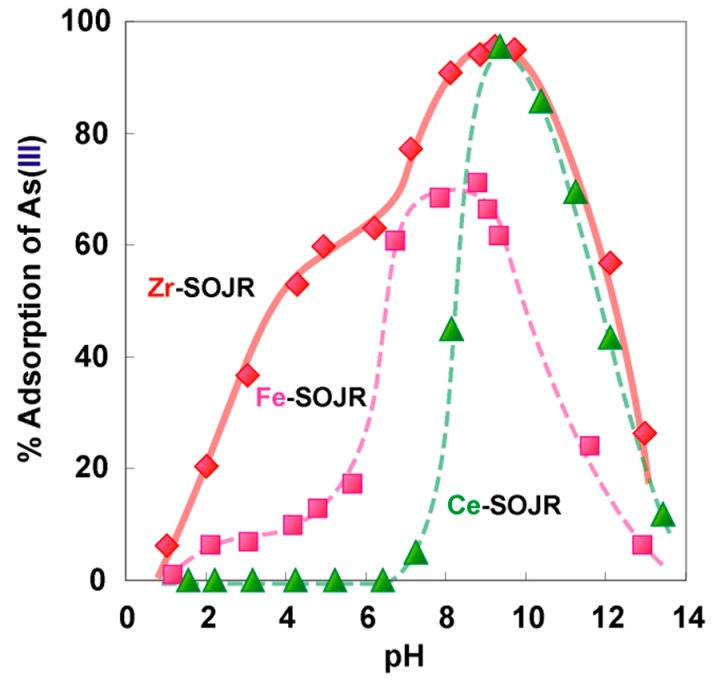
% adsorption of As(III) by SOJR gel loaded with Fe(III), Ce(III) and Zr(IV) at different equilibrium pH values [16]. (With permission for reuse from Indonesian Chemical Society).

Figure 23 shows the plot of the % adsorption of As(III) by these 3 types of SOJR gel at different equilibrium pH values. As(III) was found to be effectively adsorbed at basic pH values, i.e., the maximum % adsorption by Fe(III)- and Ce(III)-loaded SOJR was observed at pH around 7–9 and 9–10, respectively, though the pH values at which maximum adsorption occurred were not so different as for As(V). The amount of As(III) adsorbed by the Zr(IV)-loaded SOJR was more than or almost the same as the amount adsorbed by Fe(III)- and Ce(III)-loaded SOJR. We therefore concluded that Zr(IV)-loaded SOJR gel is more suitable than other SOJR gels for removing As(III) from water through adsorption.

**Figure 24:**
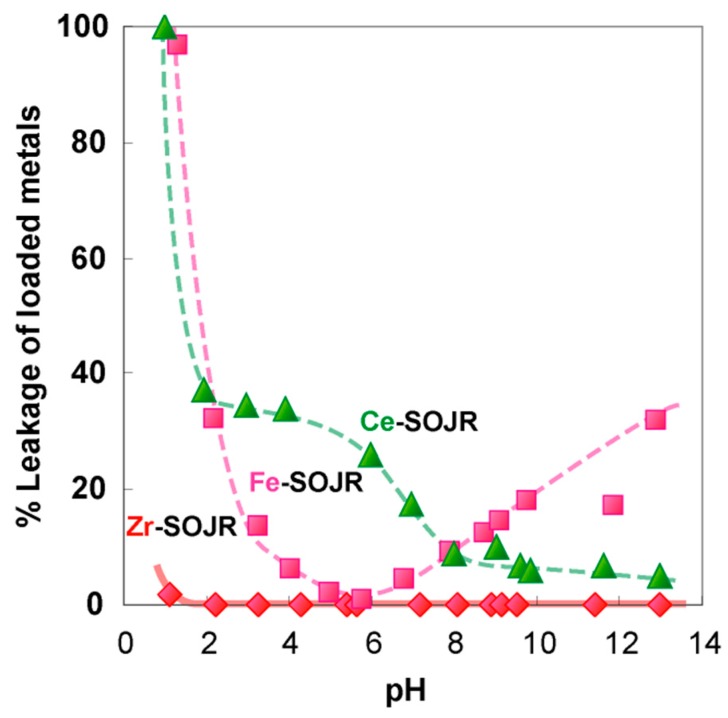
Leakage of the loaded metal ions from metal-loaded SOJR gels at different pH values [16]. (With permission for reuse from Indonesian Chemical Society).

We found that Zr(IV)-loaded SOJR gel offers another major advantage over Fe(III)- and Ce(III)-loaded SOJR gels. Figure 24 shows the plot of % leakage of the loaded metal ions from the three types of metal-loaded SOJR gels at different pH values. It can be seen that Fe(III)- and Ce(III)-loaded SOJR suffered from the leakage of loaded metal ions over a wide pH range, especially at pH values lower than 2. In contrast, negligible Zr(IV) leaked from the Zr(IV)-loaded SOJR over the whole pH range that was tested, i.e., Zr(IV)-loaded SOJR gel is free from the problem of the leakage of loaded metal ion.

Similar to the case for crosslinked pectic acid gel, adsorption of both As(III) and As(V) by Zr(IV)-loaded SOJR gel was not influenced by high concentrations of other anionic species, such as sulfate, that usually coexist with arsenic species; i.e., Zr(IV)-loaded SOJR gel is highly selective for As(III) and As(V) when other anionic species are present in water.

Both As(III) and As(V) adsorbed to Zr(IV)-loaded SOJR were easily desorbed or eluted by a high pH aqueous solution, as was expected from the results shown in Figure 22 and Figure 23.

**Scheme 9:**
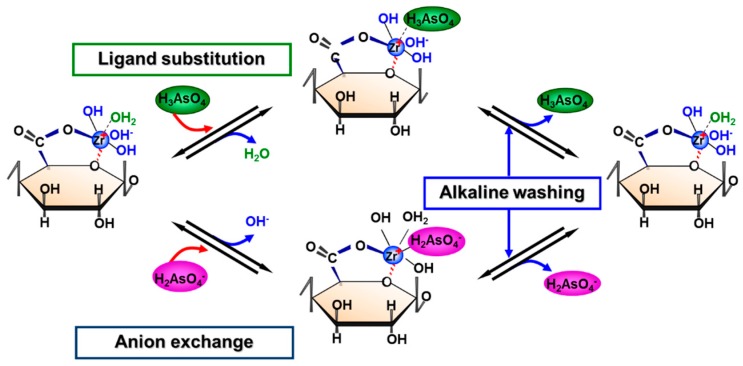
Inferred mechanisms of the adsorption and desorption of As(V) on Zr-loaded SOJR gel [16]. (With permission for reuse from Indonesian Chemical Society).

Scheme 9 shows the inferred mechanism through which As(V) was adsorbed by and desorbed from Zr(IV)-loaded SOJR gel. We assumed that the As(III) mechanisms were the same.

**Figure 25:**
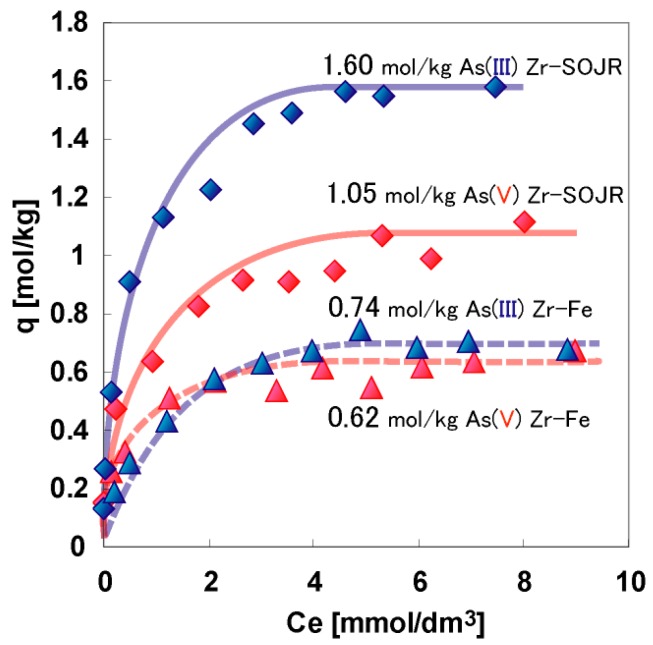
Isotherms for the adsorption of As(III) and As(V) by Zr(IV)-loaded SOJR and by zirconium ferrite (Zr-Fe) at 30 °C. Adsorbent mass = 25 mg, test solution volume = 15 mL, adsorption pH for As(V) = pH 3, adsorption pH for As(III) = pH 10 [16]. (With permission for reuse from Indonesian Chemical Society).

Figure 25 shows isotherms for the adsorption of As(III) and As(V) by Zr(IV)-loaded SOJR gel and by zirconium ferrite (ZrFe_2_(OH)_8_), a commercially available inorganic adsorbent for phosphorus. It can be seen that both As(V) and As(III) were adsorbed by both adsorbents following the Langmuir-type adsorption isotherms. The maximum As(III) and As(V) adsorption capacities of the Zr(IV)-loaded SOJR gel, determined from the isotherms, were 1.60 and 1.05 mol/kg, respectively, and the maximum As(III) and As(V) adsorption capacities of zirconium ferrite were 0.74 and 0.62 mol/kg, respectively. It was clear that SOJR biosorbent prepared from biomass waste had a higher adsorption capacity and better adsorption characteristics than the commercially available inorganic adsorbent. It is difficult to directly compare the characteristics of the biosobents and other adsorbents because different experimental conditions were used for the different sorbents, but we found that the maximum adsorption capacities of our biosorbents were somewhat higher than the maximum adsorption capacities of the other adsorbents listed in Table 1.

The removal of Sb(III and V) through adsorption to Zr(IV)- and Fe(III)-loaded SOJR was investigated in a similar way to the method described above [27].

**Figure 26:**
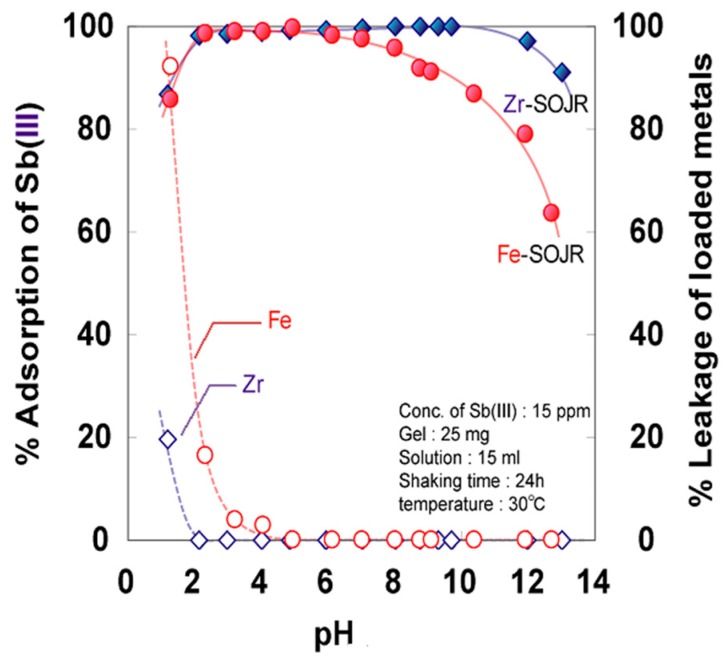
Effect of the equilibrium pH on Sb(III) adsorption by the Fe(III)- and Zr(IV)-loaded SOJR and on leakage of the loaded metal ions.

**Figure 27:**
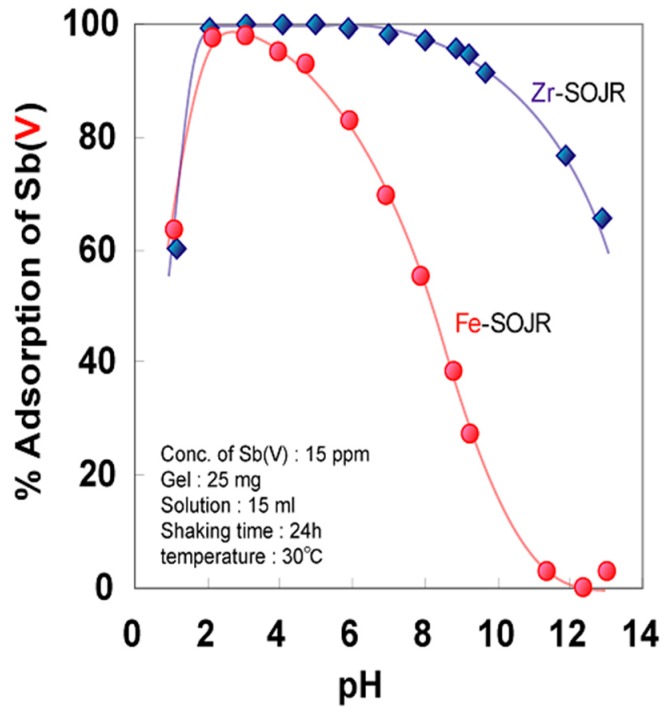
Effect of the equilibrium pH on Sb(V) adsorption by Fe(III)- and Zr(IV)-loaded SOJR [27]. (With permission for reuse from Elsevier B.V.).

Figure 26 shows % adsorption of Sb(III) from synthetic solutions by Zr(IV)- and Fe(III)-loaded SOJR gels and % leakages of the loaded metals at different equilibrium pH values. Figure 27 shows % adsorption of Sb(V) under the same conditions. It can be seen that both Sb(V) and Sb(III) were almost quantitatively adsorbed over a wider pH range by Zr(IV)-loaded SOJR gel than by Fe(III)-loaded SOJR gel. We concluded that this was related to the hydration behaviors of Zr(IV) and Fe(III) ions. The leakage of the loaded Zr(IV) was much less than Fe(III) from the SOJR gel at low pH range.

**Figure 28:**
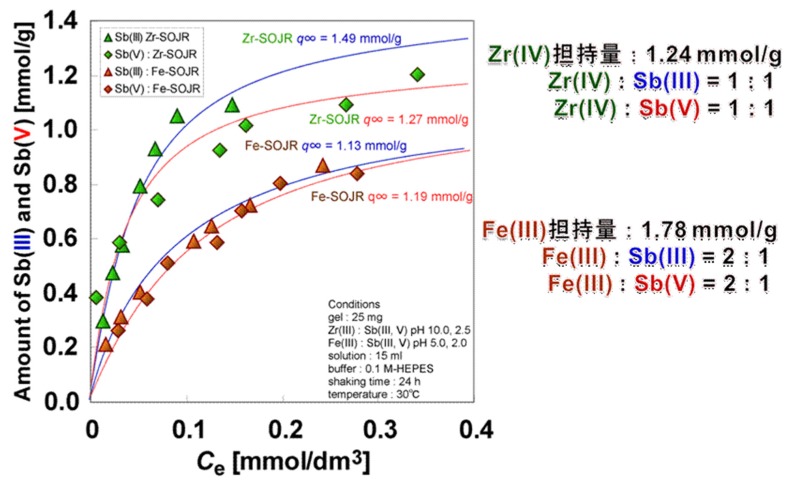
Sb(III) and Sb(V) adsorption isotherms for Zr(IV) and Fe(III)-loaded SOJR gels at 30 °C.

Similar to the adsorption of As(III) and As(V) by multivalent-metal-loaded SOJR gel, the adsorption of Sb(V and III) was unaffected by high concentration of other anionic species, such as sulfate; i.e., the biosorbent was highly selective for Sb(III) and Sb(V) over other anionic species in water. Figure 28 shows isotherms for the adsorption of Sb(III) and Sb(V) by Zr(IV)- and Fe(III)-loaded SOJR gels. The Sb(III) and Sb(V) adsorption isotherms were analyzed using the Langmuir model to determine the maximum adsorption capacities of the biosorbent for Sb. Table 2 shows the maximum Sb(III) and Sb(V) adsorption capacities of both gels and of adsorbents investigated in previous studies. It can be seen that both gels had higher Sb(III) and Sb(V) adsorption capacities than have been found for other adsorbents.

**Figure 29:**
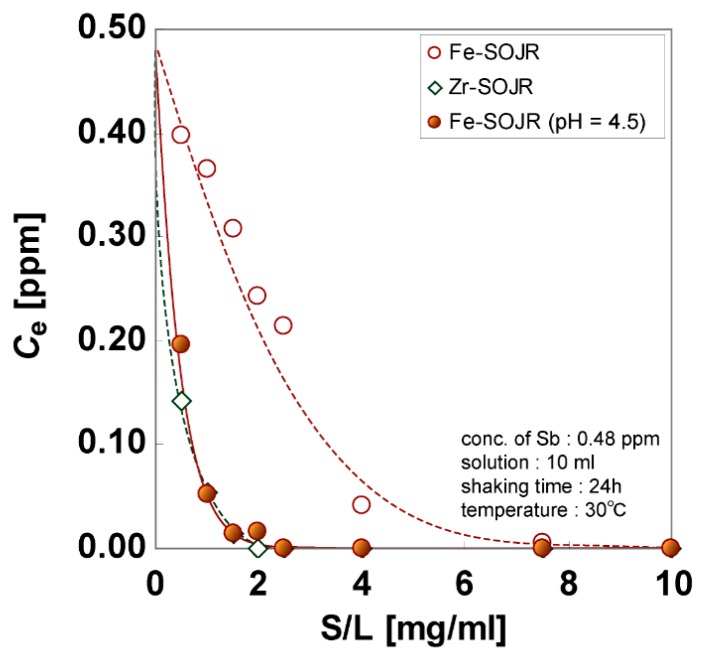
Effect of solid/liquid ratio (= dry weight adsorbent added/ volume of solution) on the residual Sb concentration when metal-loaded SOJR gels were used to remove Sb from a real waste solution (pH = 10.5) generated in a polyester fiber production factory.

The results of the fundamental adsorption tests mentioned above led us to investigate the removal of trace concentrations of Sb from a real waste solution generated in a polyester fiber production factory using Zr(IV)- and Fe(III)-loaded SOJR. Figure 29 shows the plot of the Sb concentrations remaining after the waste solution had been treated using Zr(IV)- or Fe(III)-loaded SOJR against the solid/liquid ratio. The Sb was completely removed from the waste solution at the native pH (=10.5) by Zr(IV)-loaded SOJR at a solid/liquid ratio of around 2, but a solid/liquid ratio of almost 8 was required to completely remove the Sb using the Fe(III)-loaded SOJR, though this was achieved at a solid/liquid ratio of 2 when the pH was decreased to pH 4.5.

**Figure 30:**
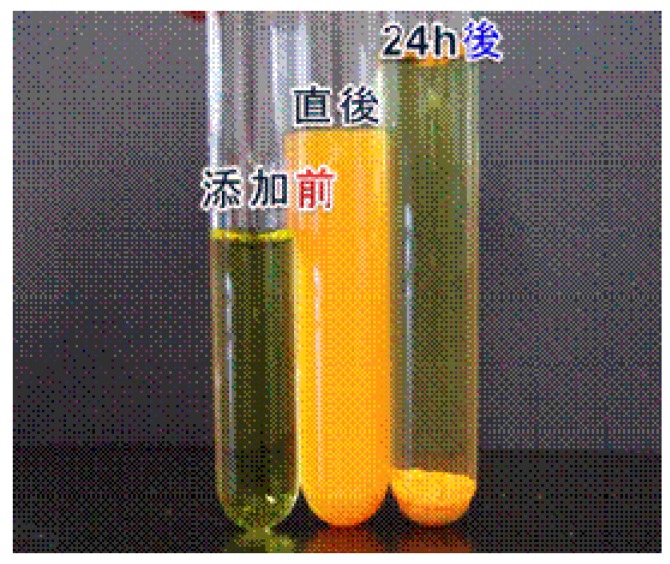
Photographs of the antimony sulfide precipitate formed when a trace Na_2_S was added to a solution of Sb(III) and Zr(IV) eluted from Zr(IV)-loaded SOJR gel using 1 M HCl. Left: before Na_2_S was added, center: just after Na_2_S was added, right: 24 h after Na_2_S was added. [27]. (With permission for reuse from Elsevier B.V.).

However, it was difficult to satisfactorily elute the adsorbed Sb(III and V) using NaOH solutions at various concentrations, unlike for As(III and V). Only 50–60% of the Sb(III) and Sb(V) was eluted using 0.1–5 M NaOH solution. We therefore attempted to desorb Sb(III and V) together with the loaded metal ions using an acidic solution then to selectively separate the desorbed Sb(III and V) from the loaded metal ions by precipitating Sb(III and V) sulfide by adding a trace concentration of NaHS or Na_2_S. Figure 30 shows an example of the formation of Sb sulfide caused by adding trace concentration of Na_2_S to a solution containing Sb(III) eluted from Zr(IV)-loaded SOJR using 2 M HCl. It can be seen that the desorbed Sb(III) was selectively recovered as a yellow precipitate of Sb(III)_2_S_3_. The method also worked for Sb(V) eluted from Fe(III)-loaded SOJR. The changes in the concentrations of Sb(III) and Fe(III) were measured before and after the addition of small volume of Na_2_S solution to the elute from the Fe(III)-loaded SOJR using 2 M HCl solution. It was found that, by adding Na_2_S solution, the concentration of Sb(III) was dramatically decreased from 810 mg/L down to 14 mg/L while the decrease in the Fe(III) concentration was nearly negligible; i.e., Sb(III) was selectively precipitated by adding trace concentration of Na_2_S. Similar phenomena were observed for Zr(IV)-loaded SOJR gel and for Sb(V).

**Figure 31:**
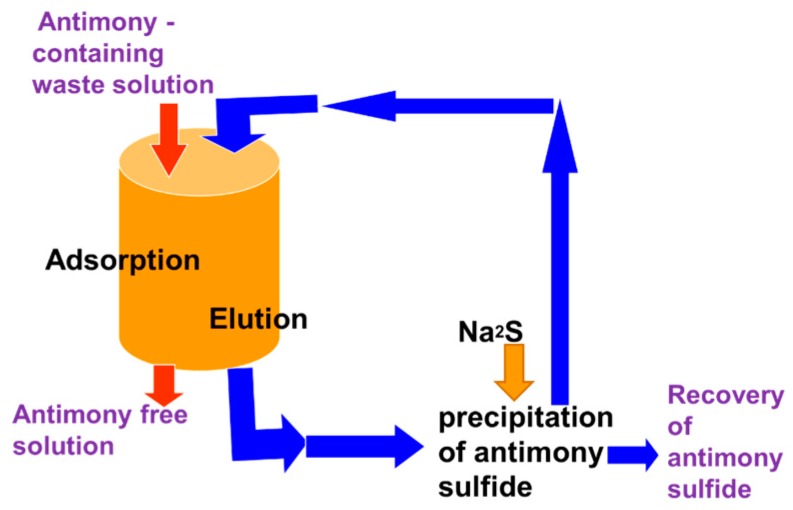
Flowchart showing the recommendation for recovering Sb from waste solutions using SOJR gel.

The results described above led us to develop the recommendations for recovering and removing Sb using metal-loaded SOJR gels shown as a flowchart in Figure 31.

## 6. Biosorbents Prepared from Seaweed

Other types of biosorbent were prepared from three kinds of seaweed containing large amounts of alginic acid [32,33] following a similar concept to that used to prepare biosorbents from orange and apple juice residues. Roughly speaking, seaweeds are classified into three groups, brown algae, green algae, and red algae. *Laminaria japonica*, *Ulva japonica*, and *Porphyra yezoensis* were used as representative brown, green, and red algae, respectively. We prepared three biosorbents from these representative seaweeds.

Some kinds of seaweeds have recently caused serious environmental problems. For example, *Ulva japonica* is now multiplying quickly in coastal areas close to large Japanese cities, and this has been attributed to increases in phosphorus and nitrogen concentrations in effluents in these areas. In summer, large amounts of these seaweeds decay on beaches along these coasts, generating bad odors and polluting the beaches. Treating these seaweeds has become a serious and refractory environmental problem for the municipalities in these coastal areas. Effective uses of seaweed waste are therefore of great interest in Japan. Preparing biosorbents from seaweed waste, as we attempted, is therefore of great interest.

**Scheme 10:**
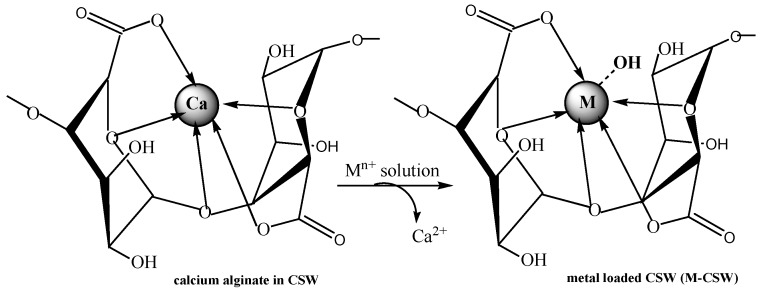
Chemical structures of calcium alginate in seaweed and metal-loaded alginic acid.

Alginic acid is present in seaweed as the Ca(II) salt, and has the egg-box structure shown in Scheme 10. Heavy metal ions coming into contact with the Ca(II) salt of alginic acid can replace the Ca(II) ions.

**Figure 32:**
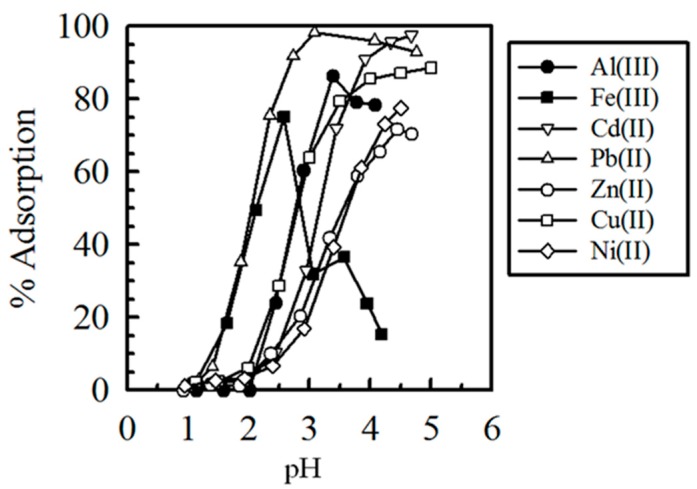
% adsorption of some metal ions by *Laminaria japonica* gel cross-linked with epichlorohydrin at different pH values.

Figure 32 shows the plots of % adsorption of some metal cations by the *Laminaria japonica* gel crosslinked with epichlorohydrin vs. the equilibrium pH values. Crosslinked alginic acid and SOJR had similar adsorption behaviors except for Fe(III) and Al(III), the adsorption of which increased with increasing pH in a similar way to the adsorption of the other metal ions only at pH values less than 2.5 and 3, respectively, but rapidly decreased with increasing pH at higher pH values. This could have been because of the formation of hydroxide precipitates of these metals, which were hardly adsorbed by the gel. Similar behaviors were found also for the gels prepared from *Ulva japonica* and *Porphyra yezoensis*.

Adsorption isotherms for Pb(II), Cd(II), La(III) and Ce(III) for the three seaweed gels were obtained at 30 °C. All the metal ions were adsorbed according to the Langmuir-type adsorption, similar to the case for SOJR. The maximum loading capacities of the metal ions on the three kinds of seaweed gels are summarized in Table 3. As can be seen, the maximum loading capacities for all the metal ions that were tested decreased in the order, *Laminaria japonica* gel > *Ulva japonica* gel > *Porphyra yezoensis* gel. The maximum loading capacities were lower for trivalent metal ions than for divalent metal ions for all of the seaweed gels, possibly because more carboxylic functional groups are required to form the 1:3 metal ion:ligand complexes of trivalent metal ions than to form 1:2 metal ion:ligand complexes of divalent metal ions. The maximum Pb(II) adsorption capacities were lower for the seaweed gels than SOJR gel, but the maximum Cd(II) adsorption capacities of *Laminaria japonica* gel and SOJR gel were the same.

**Figure 33:**
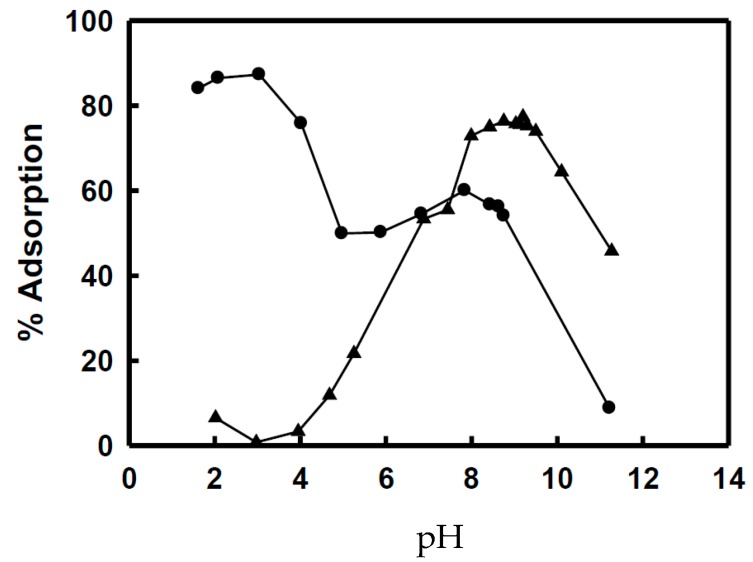
% adsorption of As(III and V) ions by Fe(III)-loaded *Laminaria japonica* gel crosslinked with epichlorohydrin at different pH values at equilibrium. ● As(V), ▲: As(III).

Figure 33 shows the plots of % adsorption of As(III and V) ions by Fe(III)-loaded *Laminaria japonica* gel against the equilibrium pH values. The adsorption behaviors were similar to the results for Fe(III)-loaded SOJR gel shown in Figure 22 and Figure 23.

## 7. Adsorptive Removal of Hexa-Valent Cr Using Biosorbents Prepared from Persimmon Extract Residue and Grape Juice Residues

**Scheme 11:**
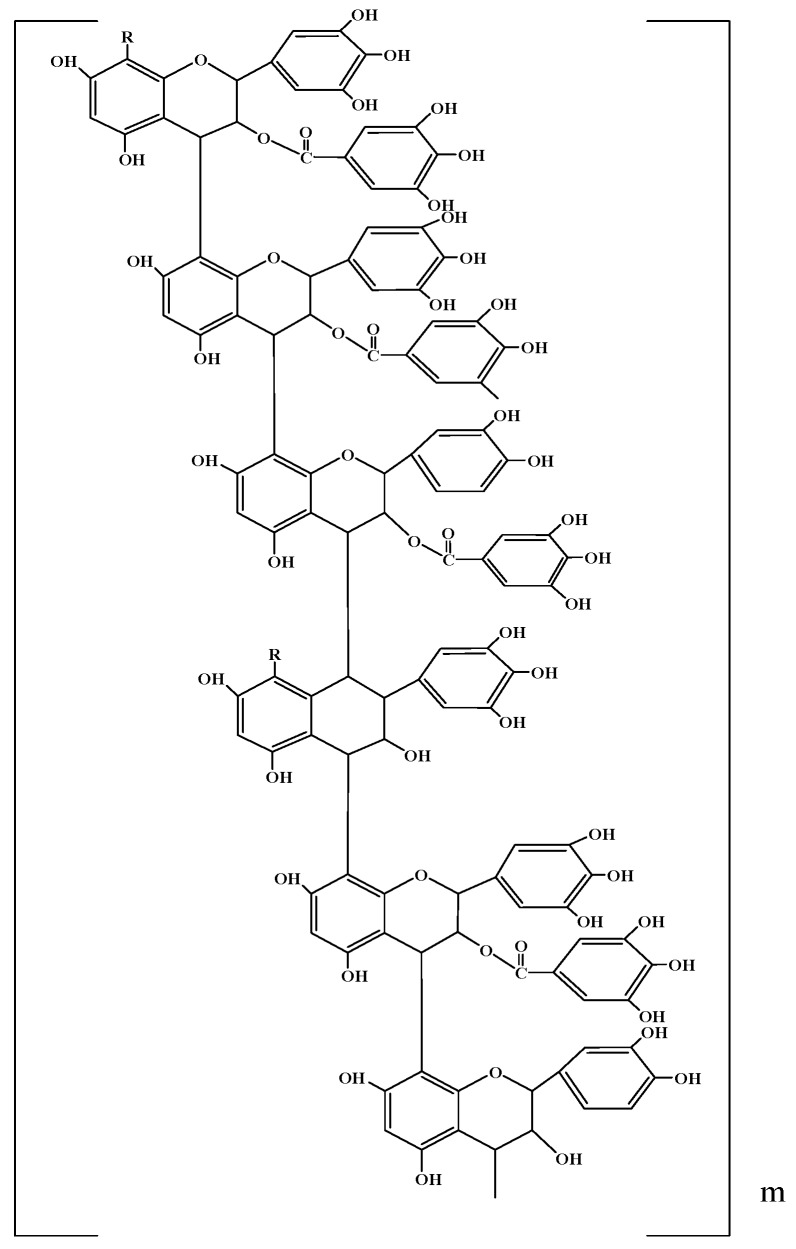
Representative chemical structure of persimmon tannin [34].

Persimmon, a popular fruit in East Asian countries such as China, Korea, and Japan, contains a polyphenol compound called persimmon tannin. This compound has a complicated chemical structure that contains many catechol and pyrogallol functional groups, as is shown in Scheme 11 [34]. The persimmon tannin content depends on the type of persimmon and the ripeness of the fruit. Persimmon extract, the juice of astringent persimmon, contains large quantities of persimmon tannin and has been traditionally used in various applications, including as a natural paint, dye, leather tanning agent, and protein-coagulating agent. Grapes are a major fruit crop around the world. Grapes contain polyphenol compounds such as anthocyanin and resveratrol. About 80% of harvested grapes are used to produce wine, and the grape waste (grape juice residue) is 20% of the weight of processed grapes. We recently prepared several novel environmentally benign biosorbents from persimmon extract powder and residue after extracting persimmon tannin extract as well as grape wastes. We expected these biosorbents could be used to recover valuable metals and remove hazardous metals from water. Here, we describe the adsorptive removal of hexa-valent Cr, a typical hazardous heavy metal, using biosorbents prepared from persimmon tannin extract residue [35] and grape waste [36].

**Scheme 12:**
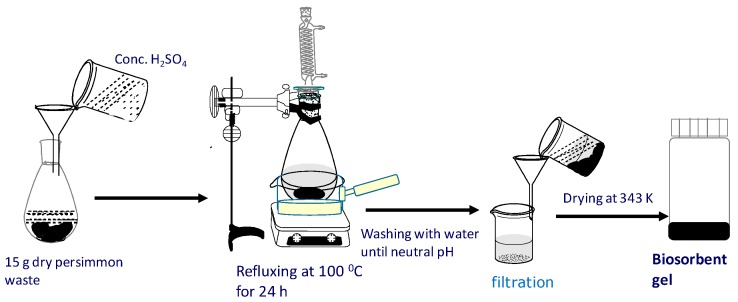
Preparation of crosslinked persimmon gel.

Both biosorbents were prepared simply by crosslinking the polyphenol functional components with other major components, such as cellulose, by refluxing the residues in concentrated sulfuric acid at 100 °C as shown in Scheme 12. The biosorbents are abbreviated as crosslinked persimmon gel and crosslinked grape gel, hereafter.

**Figure 34:**
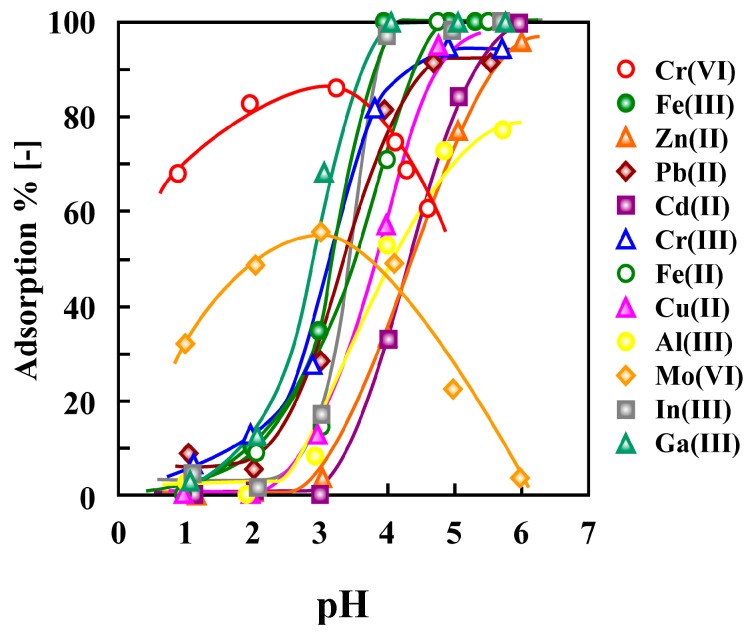
Adsorption of some metal ions from a HEPES buffer solution by crosslinked persimmon gel at different equilibrium pH values. Initial concentration of each metal = 0.5 mM, weight of gel = 10 mg, test solution volume = 10 mL, shaking time = 24 h.

Cr(VI) exists in an aqueous solution as the oxo-anionic species such as HCrO_4_^−^, H_2_CrO_4_, and CrO_4_^2−^ depending on the pH and total Cr concentration. Figure 34 shows the plot of % adsorption of some metal ions, including Cr(VI) and Cr(III) (another Cr species that is common in environmental media), by crosslinked persimmon gel at different equilibrium pH values. Except for Cr(VI) and Mo(VI), the adsorption of all metal ions existing as cationic species monotonously increased with increasing pH, suggesting that adsorption of these metal ions occurs through cation exchange as shown below, similar to adsorption by the lignocatechol gel.


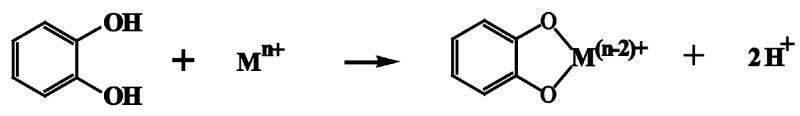


The adsorptions of Cr(VI) and Mo(VI) increased with increasing pH at low pH values but decreased with increasing pH at higher pH values, suggesting that these ions are adsorbed through another mechanism.

The aqueous chemistry of Mo(VI) is complicated. The dominant species at low pH values is the cation MoO_2_^2+^, which would have been adsorbed through cation exchange similar to the other metal cations. However, anionic species such as MoO_4_^2−^ are dominant at high pH values, so adsorption will be suppressed.

**Figure 35:**
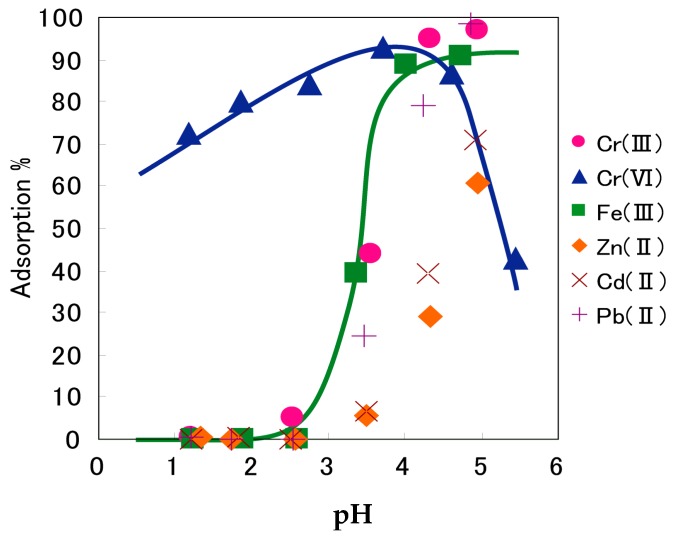
Adsorption of some metal ions from a HEPES buffer solution by crosslinked grape gel at different equilibrium pH values [36]. (With permission for reuse from Elsevier B.V.).

Figure 35 shows the similar plots for the adsorption by crosslinked grape gel. Similar adsorption behaviors were found to those found for crosslinked persimmon gel (shown in Figure 34), i.e., the adsorptions of Cr(VI) increased with increasing pH at low pH values but decreased with increasing pH at higher pH values. According to Nakajima and Baba [37], Cr(VI) is adsorbed from acidic solutions by crosslinked persimmon extract gels through esterification reaction shown below.


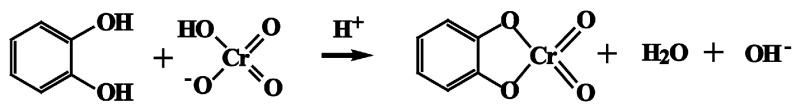


The reaction mechanism offers a reasonable interpretation of the decrease in the adsorption at high pH values (at pH higher than 3). However, the decrease in adsorption at low pH values (at pH lower than 3), is probably caused by the reduction of Cr(VI) to Cr(III), which will be hardly adsorbed at low pH values as shown in Figure 34 and Figure 35, as described below.

CrO_4_^2−^ + 8 H^+^ + 3 e^−^→ Cr^3+^ + 4 H_2_O

The reduction reaction was elucidated by investigating the adsorption kinetics of the system.

**Figure 36:**
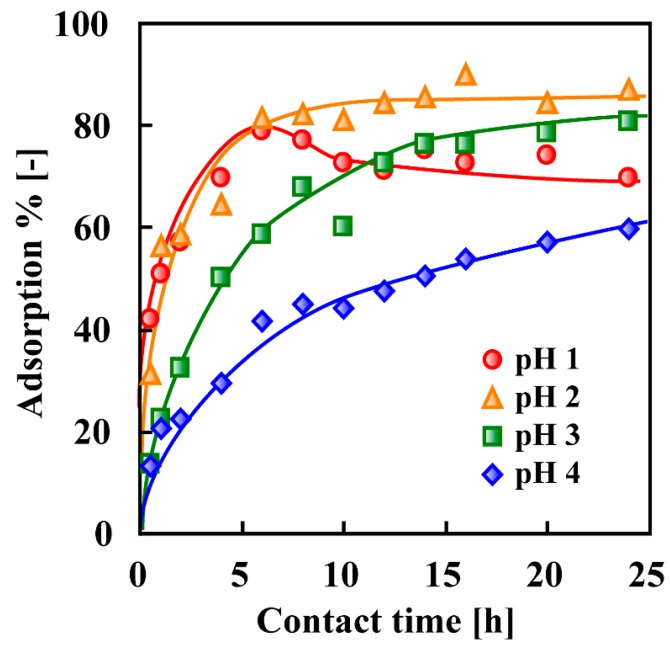
Temporal change in the % adsorption of Cr(VI) by crosslinked persimmon gel at different pH values at 30 °C. Initial Cr(VI) concentration = 13 mM, weight of gel = 10 mg, test solution volume = 10 mL [35]. (With permission for reuse from Elsevier B.V.).

**Figure 37:**
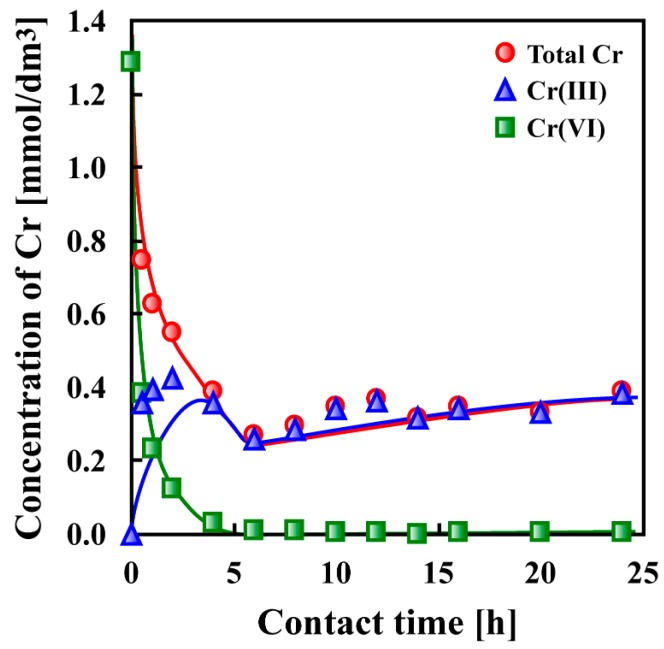
Temporal change in the Cr(VI), Cr(III), and total Cr concentrations at pH = 1 under the same conditions as shown for Figure 36 [35]. (With permission for reuse from Elsevier B.V.).

Figure 36 shows temporal change in the % adsorption of Cr(VI) by crosslinked persimmon gel at different pH values. As can be seen, the adsorption increased continually as the shaking time increased until equilibrium (determined by the pH) was reached. However, at pH = 1, the amount adsorbed increased initially but then slightly decreased and tended toward a constant value. A similar phenomenon was found for the adsorption of Cr(VI) by crosslinked grape gel [36]. Consequently, temporal changes in the total Cr, Cr(VI), and Cr(III) concentrations were found at pH = 1 when adsorption occurred from a solution containing only Cr(VI) as shown in Figure 37.

It can be seen from Figure 37 that the Cr(VI) concentration decreased continually to zero but the formation of Cr(III) caused by the reduction of Cr(VI) started just after contact was initiated. However, the Cr(III) concentration changed in a complicated way, which we attributed to the release of Cr(III) formed on the gel surface into the solution then small amount of Cr(III) being adsorbed. The driving force for the reduction reaction may be the supply of electrons from the many aromatic rings in the persimmon tannin molecules.

**Figure 38:**
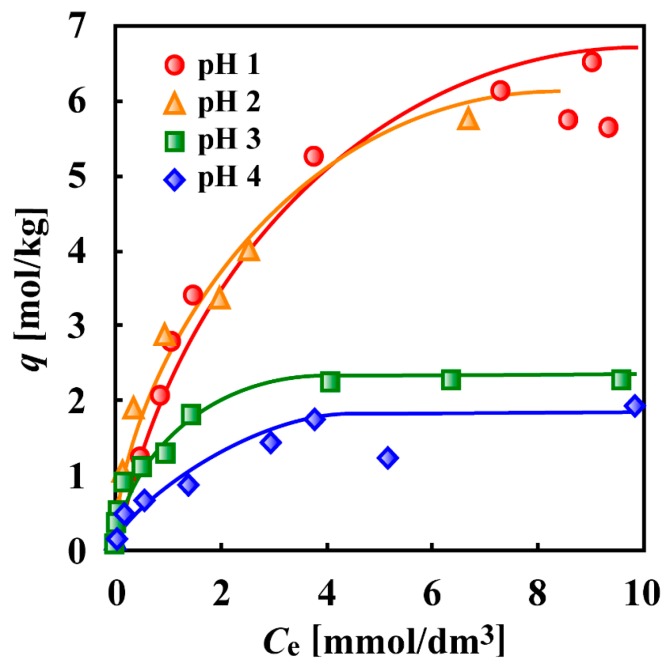
Cr(VI) adsorption isotherms for crosslinked persimmon gel at different pH values at 30 °C. Weight of gel = 10 mg, test solution volume = 10 mL, shaking time = 24 h [35]. (With permission for reuse from Elsevier B.V.).

Figure 38 shows isotherms for the adsorption of Cr(VI) by crosslinked persimmon gel at 30 °C at different pH values. It can be seen that adsorption by this biosorbent followed the Langmuir type adsorption model similar to the other biosorption systems.

These adsorption isotherms were used to calculate the maximum adsorption capacity of the biosorbent for Cr(VI). The maximum adsorption capacities at pH 1, 2, 3, and 4 were 7.18, 6.11, 2.38 and 1.96 mol/kg, respectively. Table 4 shows the maximum Cr(VI) adsorption capacities for other biosorbents and for this biosorbent at pH = 1. Cr(VI) adsorption is strongly pH-dependent and the same pH values were not used in each adsorption test, so it is difficult to compare the values, but the maximum Cr(VI) adsorption capacity of crosslinked persimmon gel was much higher than the maximum Cr(VI) adsorption capacities of other biosorbents.

**Figure 39:**
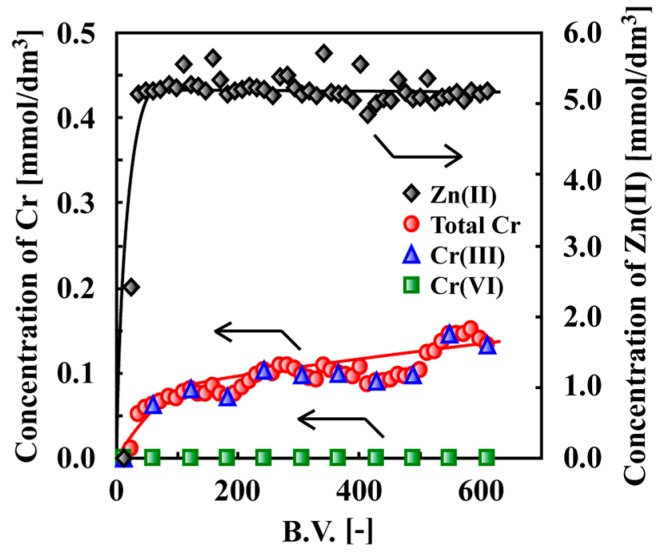
Zn(II) and Cr species breakthrough profiles for a column packed with crosslinked persimmon gel at pH = 1. Weight of packed gel = 150 mg, feed concentration of Cr(VI) = 0.5 mM, that of Zn(II) = 5 mM, flow rate = 6.5 mL/h [35]. (With permission for reuse from Elsevier B.V.).

**Figure 40:**
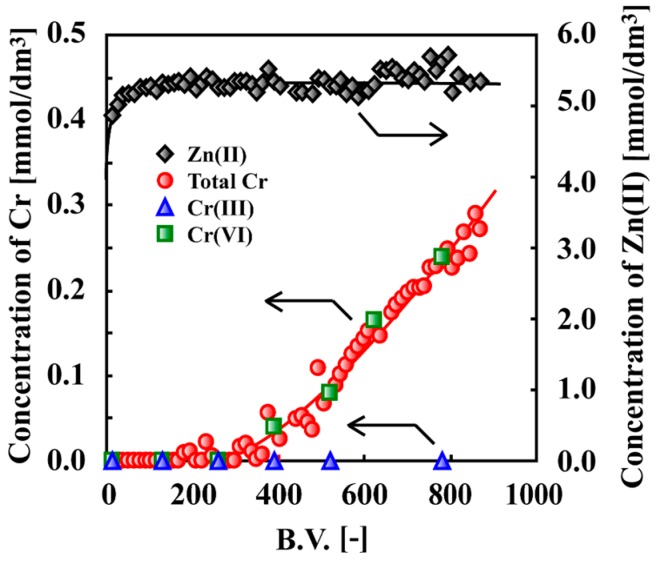
Zn(II) and Cr species breakthrough profiles for a column packed with crosslinked persimmon gel at pH = 4 using the same conditions given for Figure 39 [35]. (With permission for reuse from Elsevier B.V.).

The results of the batch-wise adsorption tests mentioned above led us to attempt to separate Cr(VI) from Zn(II) using a column packed with crosslinked persimmon gel. Figure 39 and Figure 40 show the Cr(VI), Cr(III), total Cr, and Zn(II) breakthrough profiles at pH = 1 and 4, respectively. It can be seen that Zn(II) breakthrough occurred immediately after the test solution was applied but that Cr(VI) breakthrough occurred later, suggesting that Cr(VI) and Zn(II) could be easily separated using crosslinked persimmon gel. In Figure 39, some Cr(III) leaked from the column when the test solution was applied at pH = 1, but no Cr(VI) was detected in the outlet solution. Traces of Cr(VI) can therefore be completely separated from high Zn(II) concentrations using a column packed with crosslinked persimmon gel.

It was found to be difficult to desorb or elute the adsorbed Cr(VI) from these biosorbents by simply contacting with high or low pH aqueous solutions as the cases of other biosorbents for Pb(II) and As(III and V). However, it is easily recover Cr by incinerating the Cr-loaded biosorbents, which can be totally vanished at relatively low temperature (lower than 400 °C) leaving ash of chromium. That is, easy incineration or easy end of life is another advantage of biosorbents.

## 8. Conclusions

Various biosorbents were prepared, using simple methods, from various biomass wastes (saw-dust, lignin, orange and apple juice residues, waste seaweeds, and persimmon and grape waste). The biosorbents were intended to remove hazardous heavy metals and metalloids from aqueous media. The biosorbents prepared from saw-dust, lignin, orange and apple juice residues, and waste seaweeds were highly selective for Fe(III) and Pb(II) over other metal ions, particularly Zn(II), and Pb(II) was satisfactorily separated from Zn(II) using columns packed with these bioadsorbents. Hazardous oxo-anionic As(III and V) and Sb(III and V) species were selectively separated from other anionic species such as chloride, nitrate, and sulfate using the biosorbents preloaded with high-valent metal ions, such as Fe(III) and Zr(IV). The biosorbents prepared from persimmon and grape wastes selectively adsorbed Cr(VI), the chromate anion. These results indicated that these biosorbents could be used in various practical applications.

## Figures and Tables

**Table 1:** Maximum As(V and III) adsorption capacities of the biosorbents and other adsorbents.

Adsorbent	Maximum Adsorption Capacity (mol/kg)	As Species	Authors and Reference
Zr(IV)-loaded SOJR	1.05	As(V)	Biswas et al. [18]
Ce(III)-loaded SOJR	0.56	As(V)	Biswas et al. [17]
La(III)-loaded SOJR	0.56	As(V)	Biswas et al. [17]
Fe(III)-loaded POJR	0.91	As(V)	Ghimire et al. [19]
zirconium ferrite	0.62	As(V)	Inoue [16]
activated carbon	0.014	As(V)	Gupta and Chen [20]
La(III)-impregnated alumina	0.172	As(V)	Wasay et al. [21]
Ce(IV)-doped iron oxide	0.213	As(V)	Zhang et al. [22]
Zr(IV)-loaded LDA chelating resin	0.655	As(V)	Balaji et al. [23]
*Lessinia nigrescens*, an algae	0.376	As(V)	Hansen et al. [24]
Zr(IV)-loaded SOJR	1.6	As(III)	Biswas et al. [18]
Ce(III)-loaded SOJR	0.57	As(III)	Biswas et al. [17]
La(III)-loaded SOJR	0.57	As(III)	Biswas et al. [17]
Fe(III)-loaded LDA chelating resin	0.83	As(III)	Matsunaga et al. [25]
Fe(III)-loaded POJR	0.91	As(III)	Ghimire et al. [19]
Sorghum biomass	0.05	As(III)	Haque et al. [26]

**Table 2:** Maximum Sb (V and III) adsorption capacities of metal-loaded SOJR gels and other adsorbents.

Species	Adsorbent	Maximum Adsorption Capacity (mmol/g)	Authors and Reference
Sb(III)	Zr(IV)-loaded SOJR	0.94	Biswas et al. [27]
Sb(III)	Fe(III)-loaded SOJR	1.12	Biswas et al. [27]
Sb(V)	Zr(IV)-loaded SOJR	1.19	Biswas et al. [27]
Sb(V)	Fe(III)-loaded SOJR	1.19	Biswas et al. [27]
Sb(III)	silica gel immobilized with pyrogallol functional groups	0.18	Deorkar et al. [28]
Sb(III)	hydrous oxide of Mn	0.14	Thanabalasingam et al. [29]
Sb(III)	hydrous oxide of Fe	0.10	Thanabalasingam et al. [29]
Sb(V)	activated alumina of different mesh	0.051–0.774	Xu et al. [30]
Sb(V)	aluminum-loaded Shirasu zeolite	0.020	Xu et al. [30]
Sb(V)	diphenylthiocarbazone-loaded polyurethane foam	0.22	Saeed et al. [31]

**Table 3:** Maximum adsorption capacities for Pb(II), Cd(II), La(III), and Ce(III) for the biosorbents prepared from the three kinds of seaweeds. Unit: mol/kg.

Seaweeds Metal Ions	*Laminaria Japonica*	*Ulva Japonica*	*Porphyra Yezoensis*
Pb(II)	1.35	0.77	0.27
Cd(II)	1.10	0.77	0.32
La(III)	0.87	0.51	0.23
Ce(III)	0.87	0.50	0.23

**Table 4:** Maximum Cr(VI) adsorption capacities for different biosorbents.

Feed Material of the Biosorbent	pH	Maximum Adsorption Capacity (mol/kg)	Reference
Mimosa tannin	2	5.52	Nakano et al. [38]
Persimmon extract	3	5.27	Nakajima and Baba [37]
Quaternary chitosan	4.5	0.58	Spinelli et al. [39]
Sugarcane bagasse	3	1.97	Wartell and Marshal [40]
Ocimun americanum	1.5	1.6	Levankumar et al. [41]
Grape juice residue	4	1.91	Chand et al. [36]
Persimmon extract residue	1	7.18	Inoue et al. [35]
